# High Levels of miR-7-5p Potentiate Crizotinib-Induced Cytokilling and Autophagic Flux by Targeting RAF1 in NPM-ALK Positive Lymphoma Cells

**DOI:** 10.3390/cancers12102951

**Published:** 2020-10-13

**Authors:** Domenico Sorrentino, Julie Frentzel, Géraldine Mitou, Rafael B. Blasco, Avédis Torossian, Coralie Hoareau-Aveilla, Chiara Pighi, Manon Farcé, Fabienne Meggetto, Stéphane Manenti, Estelle Espinos, Roberto Chiarle, Sylvie Giuriato

**Affiliations:** 1 Cancer Research Center of Toulouse, INSERM U1037—Université Toulouse III-Paul Sabatier—CNRS ERL5294, F-31037 Toulouse, France; domenico.sorrentino@inserm.fr (D.S.); Julie.frentzel@merckgroup.com (J.F.); geraldine.mitou@orange.fr (G.M.); avedis.torossian@univ-tlse3.fr (A.T.); coralie.ha2@gmail.com (C.H.-A.); fabienne.meggetto@inserm.fr (F.M.); stephane.manenti@inserm.fr (S.M.); estelle.espinos@inserm.fr (E.E.); 2 Department of Pathology, Boston Children’s Hospital and Harvard Medical School, Boston, MA 02115, USA; Rafael.BlascoPatino@childrens.harvard.edu (R.B.B.); pighi.chiara@gmail.com (C.P.); roberto.chiarle@childrens.harvard.edu (R.C.); 3 Ligue Nationale Contre le Cancer, équipe labellisée 2016, F-31037 Toulouse, France; 4 European Research Initiative on ALK-related malignancies (ERIA), Cambridge CB2 0QQ, UK; 5 Merck Serono S.A., Department of Biotechnology Process Sciences, Route de Fenil 25, Z.I. B, 1804 Corsier-sur-Vevey, Switzerland; 6 Department of Molecular Biotechnology and Health Sciences, University of Torino, 10126 Torino, Italy; 7 Pôle Technologique du CRCT—Plateau de Cytométrie et Tri cellulaire—INSERM U1037, F-31037 Toulouse, France; manon.farce@inserm.fr; 8 TRANSAUTOPHAGY: European Network for Multidisciplinary Research and Translation of Autophagy Knowledge, COST Action CA15138, 08193 Barcelona, Spain

**Keywords:** anaplastic large cell lymphoma, NPM-ALK, autophagy, cell death, crizotinib, combined therapy, microRNA, RAF1

## Abstract

**Simple Summary:**

Anaplastic lymphoma kinase positive anaplastic large cell lymphomas are a pediatric disease, which still needs treatment improvement. Crizotinib was the first ALK-targeted inhibitor used in clinics, but relapses are now known to occur. Current research efforts indicate that combined therapies could represent a superior strategy to eradicate malignant cells and prevent tumor recurrence. Autophagy is a self-digestion cellular process, known to be induced upon diverse cancer therapies. Our present work demonstrates that the potentiation of the crizotinib-induced autophagy flux, through the serine/threonine kinase RAF1 downregulation, drives ALK+ ALCL cells to death. These results should encourage further investigations on the therapeutic modulation of autophagy in this particular cancer settings and other ALK-related malignancies.

**Abstract:**

Anaplastic lymphoma kinase positive anaplastic large cell lymphomas (ALK+ ALCL) are an aggressive pediatric disease. The therapeutic options comprise chemotherapy, which is efficient in approximately 70% of patients, and targeted therapies, such as crizotinib (an ALK tyrosine kinase inhibitor (TKI)), used in refractory/relapsed cases. Research efforts have also converged toward the development of combined therapies to improve treatment. In this context, we studied whether autophagy could be modulated to improve crizotinib therapy. Autophagy is a vesicular recycling pathway, known to be associated with either cell survival or cell death depending on the cancer and therapy. We previously demonstrated that crizotinib induced cytoprotective autophagy in ALK+ lymphoma cells and that its further intensification was associated with cell death. In line with these results, we show here that combined ALK and Rapidly Accelerated Fibrosarcoma 1 (RAF1) inhibition, using pharmacological (vemurafenib) or molecular (small interfering RNA targeting RAF1 (siRAF1) or microRNA-7-5p (miR-7-5p) mimics) strategies, also triggered autophagy and potentiated the toxicity of TKI. Mechanistically, we found that this combined therapy resulted in the decrease of the inhibitory phosphorylation on Unc-51-like kinase-1 (ULK1) (a key protein in autophagy initiation), which may account for the enforced autophagy and cytokilling effect. Altogether, our results support the development of ALK and RAF1 combined inhibition as a new therapeutic approach in ALK+ ALCL.

## 1. Introduction

Anaplastic lymphoma kinase positive anaplastic large cell lymphomas (ALK+ ALCL) are a distinct clinicopathologic non-Hodgkin’s lymphoma, primarily occurring in children and young adults [1]. They are characterized by chromosomal translocations involving the *ALK* gene with various translocation partner genes. Nucleophosmin-Anaplastic Lymphoma Kinase (NPM-ALK) is the most prominent fusion protein observed. NPM-ALK results from the t(2;5) (p23;q35) chromosomal translocation and leads to the constitutive activation of the tyrosine kinase domain, which is driving lymphomagenesis through the activation of multiple survival/proliferation pathways [2,3,4]. 

The current treatment of this lymphoma is essentially based on aggressive chemotherapy, which is not optimal as 30% of the patients relapse 5 years post-treatment, regardless of the drug cocktail used or the treatment duration [5,6]. This observation led to the development of therapies directly targeting the NPM-ALK oncoprotein. Crizotinib was the first-in-class ALK tyrosine kinase inhibitor (TKI) [7] used in clinics for ALK+ ALCL cases that were refractory to or were relapsing after chemotherapy [8]. However, the success of this TKI was hampered by the occurrence of resistance to the drug [9,10]. This motivated both the generation of a new generation of TKI inhibitors [11,12] as well as the development of diverse combined therapies [13,14] in an attempt to prevent relapses and to eradicate the malignant cells. In this context, our work focused for the last few years on studying the possible therapeutic modulations of macro-autophagy to improve crizotinib therapy [15,16,17].

Macro-autophagy (hereafter referred to as autophagy) is a highly conserved vesicular process allowing the degradation and recycling of damaged, toxic, or unnecessary cytoplasmic material within a cell [18,19]. This process comprises five successive stages, i.e., the initiation, nucleation, elongation, fusion, and degradative steps, which are all finely orchestrated by several autophagy-related (ATG) proteins [20]. The Unc-51-like kinase-1 (ULK1) protein, in particular, plays a critical role in the initiation stage, characterized by the formation of a phagophore (or pre-autophagosomal membrane), as it integrates the cell homeostasis status, mainly through AMP-activated protein kinase (AMPK)- or mammalian target of rapamycin (mTOR)-mediated serine/threonine regulatory phosphorylations on key residues [21,22]. After membrane elongation and sealing around the unwanted cytoplasmic cargo, the mature autophagosomes fuse with lysosomes to form autophagolysomes, in which the catabolic enzymes ensure the cargo degradation. 

Dysfunctions in this important cell process have been associated with the development of diverse diseases, including cancers [23]. In this particular setting, autophagy was found to exert dual roles both in tumor progression and in response to anti-cancer therapies [24,25,26]. The role of autophagy during cancer treatment is not as simple and is often described as a “double edged sword”. Autophagy can either protect tumor cells from dying, typically by blocking apoptosis induced upon treatment [26,27], or, on the contrary, excessive or persistent activation can promote tumor cell demise either by autophagic cell death [28,29] or by ensuring connections with other cell death modalities, such as apoptosis, necroptosis, or immunogenic cell death [30,31]. 

In NPM-ALK+ ALCL cell lines, we demonstrated that autophagy was induced upon NPM-ALK inactivation, and was endowed with cytoprotective functions. Indeed, its pharmacological or molecular inhibition, combined with crizotinib treatment, increased the cytokilling effect of the TKI [15]. Recently, we further demonstrated that the potentiation of crizotinib-induced autophagy flux by downregulating the Bcl2 protein (using small interfering RNA targeting Bcl2 (siBCL2) or microRNA-34a (miR-34a) mimics) resulted in increased cell death [17]. In line with this last study, we pursued our work regarding the identification of possible microRNAs and their targets that could be therapeutically modulated, in addition to crizotinib treatment to drive autophagy toward cytotoxic functions and the outcome of tumor cell death. 

MicroRNAs (miRNAs) are a family of short, single-stranded non-coding RNAs that control post-transcriptional gene silencing [32]. The deregulation of miRNA expression levels has been extensively described in cancers, including in NPM-ALK+ ALCL [33], and has also been shown to modulate the responses to therapies [34,35]. Accumulating data in the literature indicate that these miRNAs could serve as cancer biomarkers as well as cancers therapeutics [36,37]. As autophagy manipulation is known to impact the responses to cancer therapy, a strong interest in the identification of miRNAs involved in autophagy regulation occurred during recent years [38]. Researchers described that each step of the autophagy process could be regulated by specific miRNAs [39], which offers numerous opportunities to discover potent autophagy modulators.

Our present study demonstrates that miRNA-7, known primarily to harbor tumor suppressive functions in diverse cancer types [40], and Rapidly Accelerated Fibrosarcoma 1 (RAF1), one of its targets [41,42,43,44], play essential roles in NPM-ALK+ ALCL, by controlling the autophagy flux and tumor cell fate. RAF1 is a serine/threonine kinase, best known to connect RAS to the MAPK/ERK kinase/extracellular signal-regulated kinase (MEK/ERK) pathway. The inhibition of the RAF/MEK/ERK pathway has recently been shown to promote autophagy in pancreatic cancer [45]. However, to the best of our knowledge, the mechanism by which RAF1 inhibition, specifically, could induce autophagy has not been described so far. Our work indicates, for the first time, the possible phosphorylation of ULK1 on its serine757 inhibitory residue by RAF1, which opens up a new therapeutic avenue to modulate the autophagy flux in ALK+ ALCL.

Altogether, our results indicate that the development of miR-7-5p-based therapeutics, or the addition of RAF1 inhibition to crizotinib treatment, could improve, through the potentiation of autophagy, the TKI drug killing effect and could be beneficial for ALK+ ALCL patients.

## 2. Results

### 2.1. Downregulation of miR-7-5p Expression Upon NPM-ALK Inactivation

Several groups, including ours, have demonstrated that ALK-positive ALCLs are addicted to their leading oncogene [46,47]. Thus, we reasoned that upon ALK-targeted therapy, ALCL cell lines would be submitted to an important stress likely to be responsible for changes in gene expression. As miRNAs act as well-known regulators of gene expression [48], we studied their expression profiles in Karpas-299 cells treated or not for 24 h with 500 nM crizotinib, which corresponds to the plasmatic concentration measured in patients [49] using an array containing 384 miRNAs (Human miFinder 384HC miRNA PCR Array, SABiosciences (Hilden, Germany)), selected for their documented involvement in different human cancers. We observed that crizotinib treatment led to profound changes in miRNA expression profiles, with a strong trend in miRNA downregulation: we found 89 under-expressed miRNAs versus 15 over-expressed miRNAs. 

**Figure 1:**
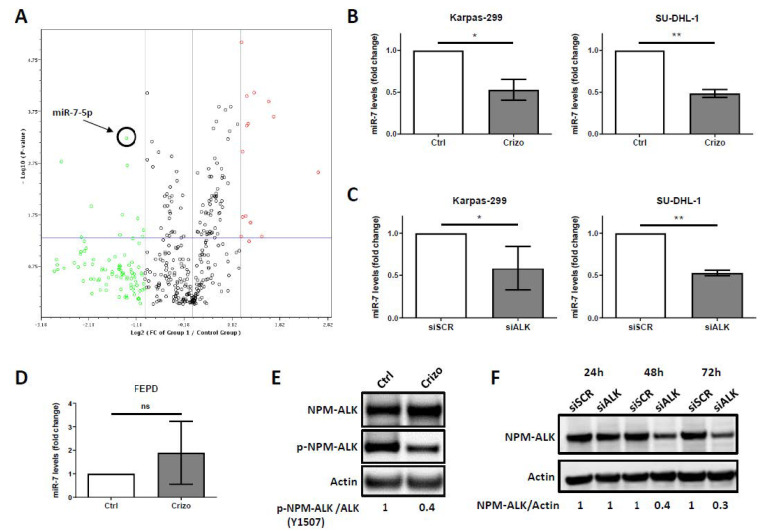
microRNA-7-5p (miR-7-5p) downregulated expression upon nucleophosmin-anaplastic lymphoma kinase (NPM-ALK) inactivation. (**A**) Karpas-299 cells were treated or not for 24 h with crizotinib (500 nM). The extracted miRNAs from control cells (*n* = 3) and from crizotinib-treated cells (*n* = 3) were submitted to a miScript miRNA PCR array (SABiosciences). The changes in miRNA expression are represented in a volcano plot. The spot corresponding to miR-7-5p is indicated. (**B**) Endogenous miR-7-5p levels were analyzed by miRNA RT-qPCR in both NPM-ALK-positive Karpas-299 and SU-DHL-1 cells and (**D**) in ALK-negative FEPD cells, untreated (Ctrl) or treated with crizotinib (500 nM, 24 h) (Crizo). (**C**) The same analysis was performed on Karpas-299 and SU-DHL-1 cells transfected with either scramble (siSCR) or ALK-targeted (siALK) siRNAs. The results are the mean of triplicates ± standard deviations (SD) from three independent experiments. They are represented in the fold regulation of the miR-7-5p levels, in comparison to the control (Ctrl) or siSCR conditions, assigned to 1. ns: not significant; * *p* ≤ 0.05; ** *p* ≤ 0.01; unpaired Student’s *t* test. (**E**) Western blot showing the total NPM-ALK, phospho-NPM-ALK in Karpas-299 cells following 24 h treatment with crizotinib (500 nM). (**F**) Western blot showing the NPM-ALK protein levels in Karpas-299 cells that were transfected for 24, 48, or 72 h either with a scramble siRNA (siSCR) or with an ALK-targeted siRNA (siALK). Actin served as the internal control to ensure equal loading.

We focused our interest on the miRNA hsa-miR-7-5p (miR-7-5p), which appeared to be the most significantly downregulated miRNA upon NPM-ALK inactivation (fold decrease: −2.6411; *p*-value = 0.0006) (Figure 1A and Table 1). We first confirmed endogenous miR-7-5p downregulation upon NPM-ALK inactivation, using pharmacological (crizotinib, Crizo) (Figure 1E) or molecular (siRNA targeting ALK, siALK) (Figure 1F) approaches, by performing miRNA RT-qPCR in two NPM-ALK-positive ALCL cell lines, Karpas-299 and SU-DHL-1 (Figure 1B,C). Of note, a 70% reduction in the NPM-ALK protein level was observed using siALK, a result consistent with our usual range of siRNA-mediated NPM-ALK knockdown (70% to 90%), as previously reported [15,16,17]. We observed that NPM-ALK inactivation reproducibly and significantly reduced miR-7-5p expression levels in the two NPM-ALK-positive ALCL cell lines. Downregulation of 47% (*p* = 0.003) and 51% (*p* < 0.0001) was observed, respectively, in the Karpas-299 and SU-DHL-1 cell lines. Of note, no significant variation of the miR-7-5p levels were observed in the ALK-negative FEPD cell line, when comparing the untreated (Ctrl) and crizotinib-treated (Crizo) conditions (Figure 1D).

### 2.2. Enhanced Levels of miR-7-5p Potentiates Crizotinib-Induced Cytotoxic Effects and Autophagic Flux

**Figure 2:**
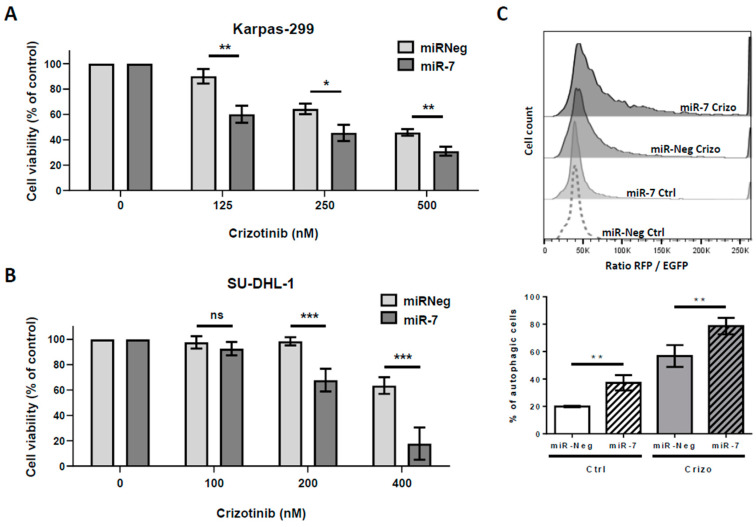
The effects of miR-7-5p increased levels on cell viability and autophagic flux. (**A**) Karpas-299 cells were transfected for 72 h with either scramble miRNA (miR-Neg) or miRNA-7-5p mimics (miR-7) and treated or not for the last 48 h with increasing doses of crizotinib (0, 125, 250, and 500 nM). The cell viability was determined using a (3-(4, 5-dimethylthiazol-2-yl)-5-(3-carboxymethoxyphenyl)-2-(4-sulfophenyl)-2H-tetrazolium, inner salt) (MTS) assay. (**B**) SU-DHL-1 cells were treated as in (**A**) with slightly different doses of crizotinib, as indicated. The graphs represent the mean values ± SD from three to four independent experiments. Statistical analysis was performed by one-way ANOVA followed by the Newman–Keuls multiple comparison test; ns: not significant; * *p* ≤ 0.05; ** *p* ≤ 0.01; *** *p* ≤ 0.001. (**C**) Karpas-299 cells stably expressing mRFP-EGFP-LC3 were transfected for 72 h with miR-7-5p mimics (miR-7) or their corresponding negative controls (miR-Neg) and treated (Crizo) or not (Ctrl) for the last 24 h with crizotinib (125 nM). Upper part: Representative FACS plots of the RFP/EGFP fluorescence ratios are shown. Bottom part: Histogram representation of enhanced autophagy flux in response to miR-7-5p transfection and crizotinib treatment. Data represent the mean ± SD; *n* = 4; ** *p* ≤ 0.01; unpaired Student’s *t* test.

As miR-7-5p was significantly downregulated in Karpas-299 and SU-DHL-1 cells submitted to NPM-ALK inactivation, we next investigated the effect of miR-7-5p enhanced levels on crizotinib-induced cytotoxic effects. Therefore, these two NPM-ALK+ ALCL cell lines were transiently transfected with either miR-7-5p mimics (miR-7-5p) or control miRNA (miR-Neg). One day after transfection, the cells were treated or not with increasing doses of crizotinib for 48 h. We observed that increased levels of miR-7-5p significantly reduced the viability of Karpas-299 and SU-DHL-1 cells treated with crizotinib and that this potentiation occurred at all tested crizotinib concentrations. As an example, crizotinib treatment at 500 nM for 48 h induced a 46 ± 2.6% decrease in Karpas-299 cells viability and the elevation of miR-7-5p levels, in the same conditions, resulted in a 69 ± 3.6% decrease in cell viability (Figure 2A). Similarly, a miR-7-5p-induced-increase of the cytotoxic effects of crizotinib was observed in SU-DHL-1 using lower doses of the drug due to the known intrinsic higher sensitivity of this cell line (Figure 2B) [50]. 

These results indicate the potentiation of crizotinib-induced cytotoxicity in ALCL cell lines by increased levels of miR-7-5p. As similar results were previously obtained by our team in modulating autophagy in combination with crizotinib treatment [17], and by other groups in other cancers types and conditions [51,52,53], we investigated whether higher levels of miR-7-5p could also impact the autophagic flux in the same way. To address this question, we used Karpas-299 cells, engineered to stably express the red fluorescent protein-enhanced green fluorescent protein-microtubule associated protein light chain 3 (RFP-EGFP-LC3) fusion protein, and measured the autophagic flux as an increase in the RFP/EGFP ratio—a method described by Gump et al. [54] that we recently validated in NPM-ALK+ ALCL [17]. 

These RFP-EGFP-LC3 Karpas-299 cells were transfected with miR-7-5p mimics or miR-Neg for 72 h and treated or not, during the last 24 h, with 500 nM crizotinib. Interestingly, we observed both a potentiation of the basal (20% ± 0.5% versus 37% ± 5.6% of autophagic cells, *p*-value = 0.0084) and crizotinib-induced autophagic flux (57% ± 8% versus 79% ± 6% of autophagic cells, *p*-value = 0.0046) in miR-7-5p transfected cells in comparison with miR-Neg transfected RFP-EGFP-LC3 Karpas-299 (Figure 2C). Similar findings were obtained when we performed endogenous LC3B immunofluorescence staining on miR-Neg or miR-7-5p transfected Karpas-299 cells, treated or not with crizotinib. 

We observed that miR-7-5p mimics increased the basal number of LC3B autophagosomal dots (2.0 ± 0.2-fold increase) and further potentiated the crizotinib-induced autophagosome formation (4.9 ± 0.5-fold increase) in comparison with control condition (Figure S1A,B). Finally, LC3B and p62 western-blotting further corroborated our findings (Figure S1C). We concluded that miR-7-5p, through the binding and downregulation of potentially several targeted mRNAs, is a potent regulator of cell viability and cell autophagy in Karpas-299 cells, as previously described in other studies [55,56,57,58].

### 2.3. miR-7-5p Directly Targets RAF1 and Downregulates Its Expression

We next investigated which of the miR-7-5p target(s) could be involved in the activation of the autophagic flux in Karpas-299 cells. To start, we looked in the literature for experimentally validated miR-7-5p targets and then narrowed our research to the ones described to participate in the regulation of the autophagy process. The *RAF1* kinase mRNA was an interesting candidate as it fulfilled these criteria both as a target of miR-7-5p, as described in melanoma cells [59], and as a regulator of autophagy, as previously reported [60,61]. 

**Figure 3:**
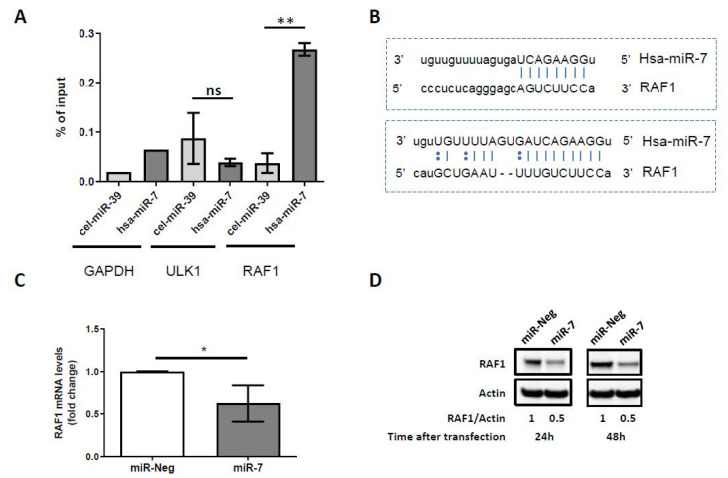
The identification of *RAF1* mRNA as a target of miR-7-5p. (**A**) The RNAs co-purified with biotinylated miR-7 versus with biotinylated cel-miR-39 (control miRNA from C. elegans) were analyzed by qRT-PCR using PCR primers for two miR-7 predicted target genes (ULK1 and RAF1). *RAF1* mRNAs were significantly pulled-down with the biotinylated miR-7 versus cel-miR-39 whereas the *ULK1* mRNAs were not. *GAPDH mRNA* was not predicted as a miR-7 target, and allowed us to evaluate and remove the pulled-down background. Data represent the mean ± SD; *n* = 3; ns: not significant; ** *p* ≤ 0.01; unpaired Student’s *t* test. (**B**) Sequence alignment between miR-7 and the 3′UTR of *RAF1* mRNA identified two complementary sites (www.microrna.org). (**C**) qPCR analysis of *RAF1* mRNA expression in control miRNA (miR-Neg) or miR-7-5p (miR-7) transfected Karpas-299 cells (mean ± SD of independent experiments, *n* = 4, * *p* ≤ 0.05; unpaired Student’s *t* test). (**D**) Representative western blot showing the RAF1 protein levels 24 or 48 h following control miRNA (miR-Neg) or miR-7-5p (miR-7) transfection in Karpas-299. Actin was used as a loading control. RAF1/Actin densitometric ratios are indicated.

Using a biotinylated miRNA pulldown assay [62,63], which allowed us to capture and identify the physical targets of miR-7-5p in untreated and crizotinib-treated Karpas-299 cells, we found selective binding of the *RAF1* mRNA to the biotinylated miRNA miR-7-5p (Figure 3A). The relevance of this result is emphasized by the presence of two predicted binding sites of miR-7-5p on the 3’ untranslated region (3’UTR) of the *RAF1* mRNA described on the microRNA.org database (http://www.microrna.org/) (Figure 3B). We validated that higher levels of miR-7-5p in Karpas-299 cells resulted in both *RAF1* mRNA and protein level decreases (Figure 3C,D). Downregulations of 37.5 ± 21.6% (*p* = 0.0395) and 50% were observed, respectively, for *RAF1* mRNA and RAF1 protein (24 or 48 h post miR-7-5p mimics transfection) in comparison with miR-Neg conditions. Similar results were obtained in SU-DHL-1 cells (Figure S2). 

Thus, consistent with other examples in the literature, our results showed a direct interaction between miR-7-5p and *RAF1* mRNA in NPM-ALK+ ALCL cells. Finally, as endogenous miR-7-5p expression levels decreased upon crizotinib treatment (Figure 1), we investigated how NPM-ALK inactivation could impact the *RAF1* mRNA and protein levels. We observed a 2 ± 0.4-fold increase (*p* = 0.0427) in the *RAF1* mRNA levels using Karpas-299 cells transfected with siRNA targeting *ALK* (siALK) (Figure S3A). This result was confirmed by analyzing the publicly available transcriptomic data, from Piva et al. and Marzec et al. [64,65] where the *RAF1* mRNA expression levels were found to be increased upon NPM-ALK doxycycline-inducible (Dox) knock down (using shRNA targeting *ALK*) (Figure S3B,C) or NPM-ALK pharmacological inhibition using pyrrolocarbazole-derived compounds (Figure S3D) or using CEP-14083 (Figure S3E). 

In accordance with these data, we found a slight and sustained increase in the *RAF1* mRNA levels upon crizotinib treatment (Figure S3F), which was corroborated by a slight but significant fold increase (1.43 ± 0.15; *p* = 0.039) in the RAF1 protein level (Figure S3G). Altogether, these results suggest that the RAF1 mRNA and protein levels are regulated, at least in part, through a NPM-ALK/miR-7-5p axis in NPM-ALK+ ALCL cells.

### 2.4. RAF1 Inhibition Mimics miR-7-5p Cellular Effects

Having demonstrated a direct interaction between miR-7-5p and *RAF1* mRNA, we next investigated whether the pharmacological inhibition of RAF1 could reproduce the results obtained by the transfection of miR-7-5p mimics on cell viability and on the autophagic flux. For that purpose, we treated the Karpas-299 cells with vemurafenib (VEM), a compound described as an ATP-competitive oral inhibitor of RAF1 and v-raf murine sarcoma viral oncogene homolog B1 (BRAF) (wild type and V600E mutated) [66,67]. The efficiency of the vemurafenib treatment was assessed by western-blotting for the decrease in the phosphorylation of the MEK proteins (Figure S4). We first analyzed the effects of miR-7-5p mimics transfection or vemurafenib alone, and the one of their combined administration on cell viability. 

As shown in Figure S5, we observed that miR-7-5p mimics alone slightly impacted the cell viability, a result consistent with other reports in the literature demonstrating the higher potential of miR-7-5p in cancer therapy when given in combination with other therapeutic agents, such as tyrosine kinase inhibitors [44]. Regarding vemurafenib treatment, we found that it impaired the viability of the fragile transfected cells, at 24 or 48 h and, importantly, that these effects were not significantly potentiated at 24 h (Figure S5, panel A) or only slightly potentiated at 48 h (Figure S5, panel B) by miR-7-5p mimics transfection. Altogether, these results indicate the stronger effect of vemurafenib, in comparison to miR-7-5p mimics alone, on cell viability, and suggest mostly common targets (likely including RAF1) upon 24 h of treatment and the appearance and combination of off-target effects at 48 h. 

**Figure 4:**
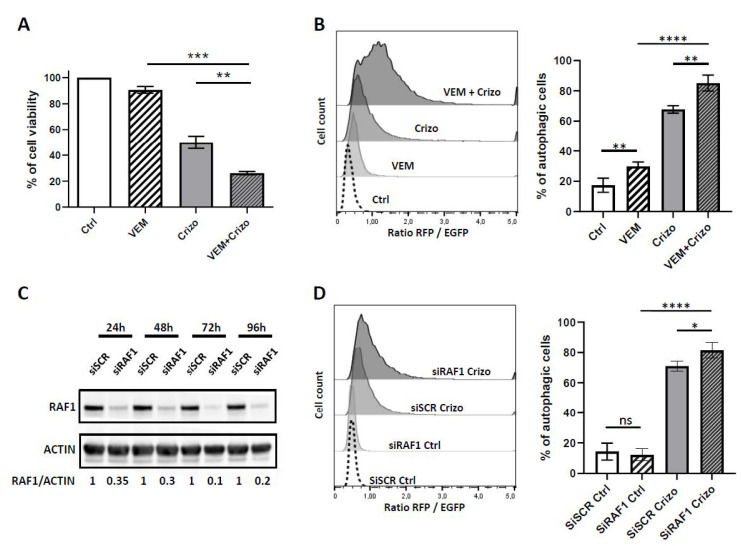
RAF1 pharmacological and molecular inactivation potentiates the crizotinib cytokilling effect and autophagy flux. (**A**) Karpas-299 cells were treated or not (Ctrl) for 48 h with crizotinib (Crizo) (500 nM) or vemurafenib (VEM) (20 µM), alone or in combination. The cell viability was determined using an MTS assay. The graphs represent the mean values ± SD from three independent experiments. Statistical analysis was performed by one-way ANOVA followed by the Newman–Keuls multiple comparison test; ** *p* ≤ 0.01; *** *p* ≤ 0.001. (**B**) mRFP-EGFP-LC3 Karpas-299 cells were treated or not (Ctrl) with crizotinib (125 nM) (Crizo) or vemurafenib (10 µM) (VEM), alone or in combination for 24 h. Left part: Representative fluorescence-activated cell sorter scan (FACS) plots of the RFP/EGFP fluorescence ratios, indicative of the autophagic flux, are shown. Right part: Histogram representation of the enhanced autophagy flux in response to crizotinib and vemurafenib treatment. Data represent mean ± SD; *n* = 4; ** *p* ≤ 0.01; **** *p* ≤ 0.0001; unpaired Student’s *t* test. (**C**) Western blot showing RAF1 protein levels in Karpas-299 cells that were transfected for 24, 48, 72, or 96 h either with a scramble siRNA (siSCR) or with an RAF1-targeted siRNA (siRAF1). Actin served as the internal control to ensure equal loading. The RAF1/Actin densitometric ratios are indicated. (**D**) mRFP-EGFP-LC3 Karpas-299 cells were transfected for 36 h with siRNA targeting RAF1 (siRAF1) or corresponding negative controls (siSCR) and treated (Crizo) or not (Ctrl) for the last 18 h with crizotinib (125 nM). Left part: Representative FACS plots of the RFP/EGFP fluorescence ratios are shown. Right part: Histogram representation of the enhanced autophagy flux in response to crizotinib treatment and RAF1 molecular knock down. Data represent the mean ± SD; *n* = 4; ns: not significant; * *p* ≤ 0.1; **** *p* ≤ 0.0001; unpaired Student’s *t* test.

We then studied the impact of crizotinib and vemurafenib, as single or combined treatment, on cell viability and autophagy flux. As shown in Figure 4A, we observed a significant decrease in cell viability, from 50% ± 5% upon single crizotinib treatment to 26% ± 1.3% upon crizotinib and vemurafenib co-treatment (*p* = 0.011). Regarding the autophagic flux, we observed that the addition of vemurafenib potentiated both the basal and crizotinib-induced autophagic flux in the RFP-EGFP-LC3 Karpas-299 cells, as assessed by the increase of the RFP/EGFP ratio (Figure 4B). These results were further confirmed by the LC3B immunofluorescence, which showed LC3B positive autophagosome formation upon crizotinib and vemurafenib single treatments (2.3 ± 0.7- and 2.3 ± 0.6-fold increases, respectively) and a potentiated autophagy flux upon combined treatment (4 ± 0.6-fold increase) in comparison with the control condition (Figure S6). 

Similarly, a slight but significant potentiation of the crizotinib-induced autophagic flux was observed upon RAF1 molecular downregulation using siRNA targeting *RAF1* (Figure 4C), in comparison with the siRNA Scramble (siSCR) condition (Figure 4D). We also noticed that there was no potentiation of the basal autophagy when using siRAF1 transfected cells, a result different than the ones observed with the pharmacological inactivation of RAF1 using vemurafenib. Thus, we propose that the vemurafenib larger spectrum of action could account for this difference in basal condition.

Finally, to understand by which means cell death was potentiated, we investigated the impact of RAF1 inhibition on apoptotic cell death. We found that the molecular downregulation of RAF1, combined with crizotinib treatment, significantly increased cell apoptosis (fold increase: 3.24 ± 0.60) in comparison with crizotinib single treatment (fold increase: 1.97 ± 0.40) (*p* = 0.0462) and normalized to the untreated condition (Figure S7). Similar results were observed upon vemurafenib and crizotinib co-treatment. Altogether, our data showed that the inhibition of RAF1 potentiated both crizotinib-induced toxicity and crizotinib-induced autophagic flux in Karpas-299 cells.

### 2.5. RAF1 Controls the Autophagic Process in Crizotinib-Treated Karpas-299 Cells through Phosphorylation of ULK1 at Serine757 Inhibitory Residue

**Figure 5:**
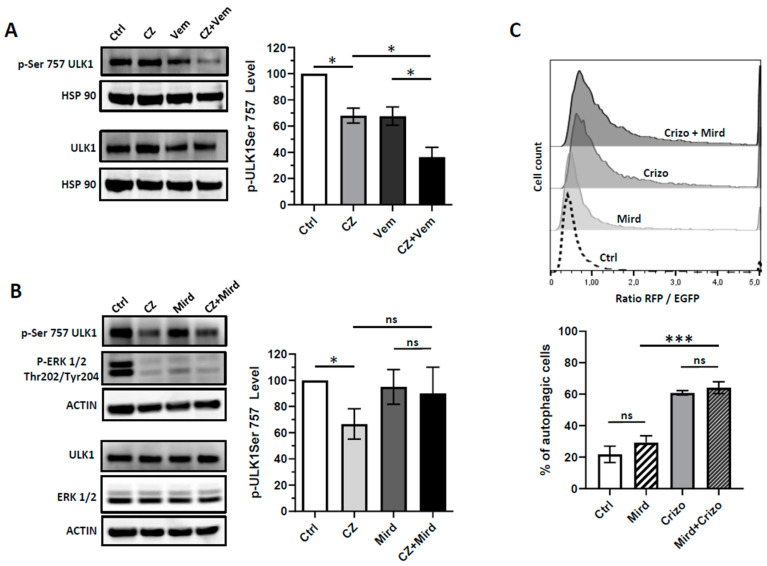
The effects of combined treatment on ULK1 Serine 757 phosphorylation. Karpas-299 cells were treated or not (Ctrl) overnight (16 h) with crizotinib (CZ) 250 nM and either vemurafenib (Vem) 10 µM (**A**) or mirdametinib (Mird) 1 µM (**B**) as single agents or in combination. Left parts: Whole cell lysates were loaded twice on a same gel and probed for total ULK1 and phospho-Ser757 ULK1 contents by western blot. The efficiency of the mirdametinib treatment was assessed by western-blotting for decreased phospho-ERK1/2. Right parts: Densitometry analysis of the phospho-ULK1 (Serine757) levels was calculated relative to the control samples (normalized over the hsp90 or actin signals) and relative to the total ULK1 signals. The data are representative of three independent experiments. (**C**) mRFP-EGFP-LC3 Karpas-299 cells were treated or not (Ctrl) with crizotinib (250 nM) (Crizo) or mirdametinib (1 µM) (Mird), alone or in combination for 24 h. Upper part: Representative FACS plots of the RFP/EGFP fluorescence ratios, indicative of the autophagic flux, are shown. Lower part: Histogram representation of the enhanced autophagy flux in response to crizotinib and mirdametinib treatment. Data represent mean ± SD; *n* = 3; ns: not significant; * *p* ≤ 0.1; *** *p* ≤ 0.001; unpaired Student’s *t* test.

Having found that RAF1 participates in the control the autophagic flux in NPM-ALK+ ALCL cell lines, we hypothesized that this effect could be attributed to its serine or threonine kinase activity on key players of the autophagy machinery. We focused our interest on ULK1 as its regulation through serine phosphorylation, notably by two major kinases controlling the initiation step, i.e., mTOR and AMPK, has been largely documented [22]. We first investigated if the pharmacological inhibition of RAF1 (using vemurafenib) could impact the phosphorylation of ULK1 at its phosphoSer757 site (inhibitory phosphorylation). As shown in Figure 5A, we found a significant decrease (63.7 ± 13%; *p* = 0.0136) of the phosphoSer757 ULK1 signal in Karpas-299 cells submitted to the combined NPM-ALK and RAF1 inactivation (using crizotinib and vemurafenib, respectively) in comparison with no treatment or single drug conditions (crizotinib: 32 ± 9.8%; *p* = 0.0315) (vemurafenib: 32.3 ± 12.1%; *p* = 0.0381). In the same line, we found a decrease in the phosphorylation of the Ser757 ULK1 residue in Karpas-299 cells knocked-down for RAF1 (upon either siRAF1 or miR-7-5p mimics transfection) and treated with crizotinib, when compared to the untreated cells (Figure S8A,B). 

This result was further corroborated using Karpas-299 clonal cells invalidated for RAF1 using the CRISPR/Cas9 system (Figure S8C,D). By combining crizotinib with the MEK inhibitor PD0325901 (Mirdametinib), we found that this ULK1 phosphorylation did not rely on the MEK/ERK kinases (Figure 5B). This result highlighted that the function of RAF1 in regulating ULK1 phosphorylation occurred independently of the MEK/ERK signaling cascade. In accordance, we found that the treatment with mirdametinib did not potentiate the basal and crizotinib-induced autophagic flux, as assessed by flow cytometry using the RFP-EGFP-LC3 Karpas-299 cells (Figure 5C). 

**Figure 6:**
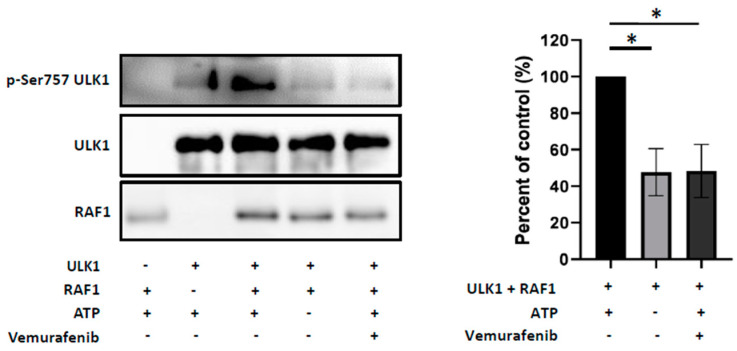
RAF1 can directly phosphorylate ULK1 at the Ser757 residue. Left part: Representative results of an in vitro kinase assay performed by incubating the human recombinant RAF1 kinase (306–648) (20 ng) and ULK1 full length (1–1050) (200 ng) proteins in the presence (+) or absence (−) of ATP (200 µM) and vemurafenib (100 nM), as indicated. RAF1 kinase activity was monitored by western blot analysis using the indicated antibodies. Right part: Histogram representation of the kinase activity of RAF1 on the serine757 site of the ULK1 protein. Data represent the mean ± SEM; *n* = 3; * *p* ≤ 0.1; unpaired Student’s *t* test.

Finally, we investigated whether RAF1 could directly phosphorylate ULK1 at Ser757 inhibitory residue and performed an in vitro kinase assay. As shown on Figure 6, we found indeed that the recombinant RAF1 enzyme phosphorylated the ULK1 protein as a substrate on its Ser757 residue. This phosphorylation was reproducibly and significantly impaired in the absence of ATP or in the presence of vemurafenib in the kinase assay buffer, where a 52.3 ± 7.4% (*p* = 0.0195) and 51.7 ± 8.4% (*p* = 0.0255) decrease in the RAF1 activity were observed, respectively. Altogether these results strongly suggest that RAF1 may negatively control the autophagy process in NPM-ALK+ ALCL cells through phosphorylation of ULK1 at Ser757 residue.

## 3. Discussion

The use of crizotinib in the treatment of ALK+ ALCL is so far limited to patients not responding to chemotherapy or who underwent relapses [68]. Its use as a front line therapy is currently being investigated in three clinical trials (NCT01979536/NCT02034981/UMIN000028075). Despite the initial successes of ALK TKI, cases of resistance to the drug have been reported [8,9,69]. To overcome or prevent these resistances, the design of combined therapeutic strategies, aiming at inhibiting the ALK oncogene concomitantly with another essential cell growth and survival pathway, appears as a promising approach [1,6,14,70]. 

In the literature, targeting the autophagic flux was described as a rational way to fight resistance in diverse hematological malignancies [71,72]. Indeed, depending on the context, autophagy inhibition [27,73] and activation [74,75,76] have been shown to prime cancer cells to cell death. In consensus with this literature, we first demonstrated that the inhibition of crizotinib-induced cytoprotective autophagy could be beneficial for the treatment of NPM-ALK positive lymphoma [15]. Recently, we further reported that combining crizotinib with BCL-2 knock-down resulted in autophagy flux potentiation and increased cell death [17].

**Figure 7:**
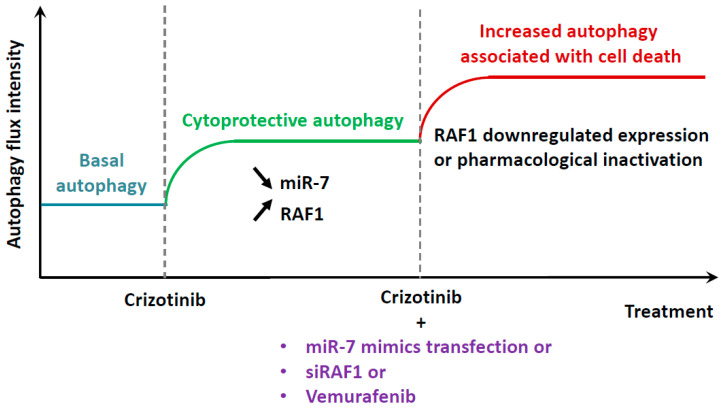
The proposed model of action of NPM-ALK and RAF1 inhibitions on autophagy flux and cells outcome. The pharmacological inactivation of the NPM-ALK oncogene in NPM-ALK+ ALCL was previously shown to induce a cytoprotective autophagic flux. This status was shown to be associated with the downregulation of miR-7-5p levels and the increased expression of one of its targets: RAF1. When pharmacological or molecular tools allowing RAF1 inactivation were used in combination with NPM-ALK inhibition, the autophagy flux was potentiated and associated with increased cell death, therefore, highlighting the superiority of the co-treatment to kill NPM-ALK+ lymphoma cells.

In the present study, we identified another possible combined therapy, associating NPM-ALK and RAF1 inhibition, which also increased autophagy and cell death (Figure 7). RAF1 inhibition was achieved using either pharmacological (vemurafenib) [66] or molecular (siRNA targeting RAF1 or miR-7-5p mimics) approaches [43]. Of translational importance, vemurafenib is already used for the treatment of diverse RAF-driven cancers [77], and miR-7-5p replacement therapy holds the promise for clinical development, but still requires further investigations [44]. 

The delivery of miRNAs in patients remains challenging, and their formulation and appropriate route of administration are the matter of abundant studies in mice models [78,79,80]. We thus analyzed the literature in search for other studies that could corroborate our in vitro findings. Of relevant interest, miR-7-5p mimics were found to potentiate the gefitinib induced cytotoxicity in lung cancer, via inhibiting the epidermal growth factor receptor (EGFR) and insulin like growth factor 1 receptor (IGF1R) pathways [81], and other studies reported that inhibiting the EGFR signaling allowed autophagy to contribute to cell death [82]. 

In the same line, Song et al. reported that radiation-induced exosomal miR-7-5p promoted bystander autophagy in non-targeted bronchial epithelial cells through inhibition of the EGFR/Akt/mTOR axis [57]. The authors proposed that this bystander autophagy induction, through exosomal miR-7-5p, might play a role in the detrimental effect of RIBE (Radiation-Induced Bystander Effects). Of interest, an enhanced and detrimental autophagy was similarly observed following an ALKBH5 silencing/miR-7-5p increase and subsequent downregulation of the EGFR/Akt/mTOR axis in epithelial ovarian cancer cells [58].

In the literature, RAF1 inhibition was shown to induce autophagy and tumor regression in a conditional mouse model for RAF1-induced lung tumorigenesis [61]. Conversely, Kinsey et al. recently demonstrated that the RAF1/MEK/ERK pathway inhibition elicited protective autophagy in pancreatic ductal adenocarcinoma cells, through the activation of the LKB1/AMPK/ULK1 signaling axis [83]. Another study showed that RAF1 was found co-localized with LC3-II at autophagosomal membranes in different cell types [84]. Altogether this literature suggests that RAF1 could possibly interact and regulate different autophagy machinery proteins. 

In line with this hypothesis, we demonstrated in an in vitro kinase assay that RAF1 was able to phosphorylate ULK1 on its serine 757 inhibitory residue and that cells submitted to the combined NPM-ALK and RAF inactivation, but not MEK inactivation, showed a strong defect in this phosphorylation. Thus, our data indicate that RAF1 may be a new actor in regulating ULK1 phosphorylation and function, a result that should motivate further studies in other models. 

We also found that RAF1 may impact the autophagic pathway independently of the MEK/ERK kinases, a result consistent with the independent functions of RAF1 and MEK/ERK proteins in NPM-ALK+ ALCL [85], as well as in other cancers [86,87]. We then investigated whether RAF1 and ULK1 could interact directly or could be part of the same signaling complex. As we had difficulties in proving such proximity between the two proteins, we hypothesized that their interaction could be transient or could require and take place at autophagosomal membranes. Thus, further investigations are needed to answer this question. 

Our work demonstrates, for the first time, that NPM-ALK and RAF1 inhibition, by potentiating autophagy and cell death, could be beneficial for ALK+ ALCL patients. Regarding the possible extension of our findings to other ALK-dependent cancers, ALK inhibition in ALK-mutated neuroblastoma [88] or ALK overexpressing rhabdomyosarcoma [89] has been proposed to activate autophagy associated with cell death. Mechanistically, researchers suggested that this effect relied mainly on the inactivation of the PI3K/Akt/mTOR signaling axis, a well-known negative regulator of the autophagy process [90]. 

Previous reports also highlighted the survival role of the mTOR pathway in NPM-ALK+ ALCL [91,92,93]. Consistently, in NPM-ALK+ ALCL [13], as in EML4-ALK lung cancers [94] and ALK-mutated neuroblastoma [95,96], a synergistic activity of ALK and mTOR inhibition had been described, although the authors did not investigate the involvement of autophagy associated with cell death in these settings. Of importance, we reported in NPM-ALK+ ALCL that such combined ALK and mTOR inhibition, using crizotinib and rapamycine, respectively, resulted in an increased autophagic flux and loss of cell viability [17]. 

In the same line, another interesting study, which was published during the time of the revision of our present manuscript, reported the elimination of dormant, autophagic and drug-resistant ovarian cancer cells upon crizotinib or other ALK inhibitors treatment through the potentiation of autophagy and subsequent increase in apoptosis [97]. Thus, it is tempting to propose, based on our studies and on the analysis of the literature, that any ways to push and/or turn autophagy into a death process in NPM-ALK+ ALCL, and potentially in other ALK-related malignancies, should improve the TKI therapy and patient outcome.

## 4. Materials and Methods 

### 4.1. Cell Lines and Cell Culture Conditions 

The Karpas-299 NPM-ALK-positive ALCL cell line bearing the t(2;5)(p23;q35) translocation was a gift from Pr C. Gambacorti-Passerini and Dr M. Ceccon (Department of Health Sciences, University of Milano-Bicocca, Milan, Italy). This cell line was cultured in Roswell Park Memorial Institute medium 1640 (RPMI-1640) supplemented with 10% Fetal Calf Serum (FCS), 2 mM L-glutamine, 1 mM sodium pyruvate and 100 U/mL penicillin/streptomycin (all from Invitrogen, Carlsbad, CA, USA) at 37 °C with 5% CO_2_, and was maintained in the exponential growth phase. The SU-DHL-1 NPM-ALK-positive ALCL cell line bearing the t(2;5)(p23;q35) translocation was obtained from DSMZ (German Collection of Microorganisms and Cell Culture, Braunschweig, Germany). The FEPD ALK-negative cell line was a gift from Dr. K. Pulford (Oxford University, Oxford, UK). SU-DHL-1 and FEPD cells were cultured in Iscove’s Modified Dulbecco’s Medium (IMDM) supplemented with 20% FCS, 2 mM L-glutamine, 1 mM sodium pyruvate, and 100 U/mL penicillin/streptomycin at 37 °C with 5% CO_2_ and were also maintained in exponential growth phase. 

### 4.2. Generation of RFP-GFP-LC3 Karpas-299 Cells

Five million Karpas-299 cells, in the exponential growth phase, were transfected by 5 µg of mRFP-EGFP-LC3 plasmid (kindly provided by Pr Tamotsu Yoshimori (Department of Genetics, Osaka university, Osaka, Japan)) by electroporation at 250 V and 950 μF using the Gene pulser II Electroporation system (BioRad, Marnes-la-Coquette, France). Stable transformants were selected in complete medium containing increasing concentrations of G418 (Sigma-Aldrich, St. Louis, MO, USA), from 0.5 mg/mL to 1.2 mg/mL. For higher sensitivity and consistency, single cell sorting for RFP and GFP double-positive cells was performed to isolate individual clones. One clone, called RFP-EGFP-LC3 Karpas 299 was then further amplified and tested for its ability to measure autophagic flux using ratiometric flow cytometry [54] upon stimulation with Crizotinib (previously described to induce autophagic flux in Karpas-299 cells, [15]). To maintain overtime, the RFP-EGFP-LC3 Karpas-299 cells selection pressure, 1.2 mg/L of G418 was added to the medium two times a week.

### 4.3. Generation of Karpas-299 Cells Knocked Down for RAF1 

We used the lentiCRISPR vector (Addgene, Cambridge, MA, USA, plasmid #52961) established by the Zhang laboratory [98]. The design of the target gRNA sequences against *Raf1* was performed using the platform: http://crispr.mit.edu/. *Raf1* exon 2 was the input for software analysis. Two top-scoring gRNA sequences were selected (gRNA#1 = AGCAGTTTGGCTATCAGCGCCGG and gRNA#2 = GCAGTTTGGCTATCAGCGCCGGG) and cloned into the lentiCRISPR vector as recommended by the Zhang Lab GeCKO website http://www.genome-engineering.org/gecko/. The resulting lentiCRISPR- *Raf1* vectors were transfected into the host packaging cell line (HEK 293T) for the production of lentivirus encoding Cas9 and the respective gRNAs. The lentivirus-containing media from HEK 293T cells were subsequently transferred onto Karpas-299 ALCL cells to generate *Raf1*-deleted clones. After 24 h of infection, the medium was replenished with complete media containing antibiotic selection puromycin (2 μg/mL). The culture medium was changed every 2 days for 2 weeks to generate stable RAF1 depleted cells. A single cell dilution allowed the selection of clones that were evaluated for RAF1 (Cell Signaling Technology #3738) expression by western blot. Only one clone (clone 22) was used in our studies.

### 4.4. Reagents

Crizotinib (Xalkori) was synthetized and purchased at @rtMolecule (Poitiers, France). Chloroquine (Aralen) (#C6628) was purchased from Sigma-Aldrich (St. Louis, MO, USA). Stock solutions of crizotinib and chloroquine were prepared in phosphate buffered saline (PBS). Vemurafenib (PLX4032) and PD0325901 (Mirdametinib) were purchased from Selleckchem (Munich, Germany), and stock solutions were prepared in DMSO. ATP was purchased from Thermo Fisher Scientific (Illkirsh-Graffenstaden, France).

### 4.5. MiRNA Expression Profiling Assay

Karpas-299 cells were treated or not for 24 h with crizotinib 500 nM, washed once with PBS, and harvested. Frozen cell pellets were sent to SABiosciences (Hilden, Germany), where both the miRNAs extractions and the miScript miRNA PCR array were performed. The amplification data (fold changes in C_t_ values of all the miRNAs) were analyzed by the ΔΔC_t_ method. The data were normalized to control (PBS-treated) cells, assigned as 1. All experiments were repeated thrice.

### 4.6. RNA Extraction

The total RNA extractions were performed using the Trizol^®^ reagent, (Invitrogen, Carlsbad, CA, USA) according to the manufacturer’s instructions. No additional purification steps were performed. 

### 4.7. Reverse Transcription

Messenger RNA (mRNA) was converted to cDNA with the Prime script RT-PCR kit (Takara Bio USA, Inc. (Mountain View, CA, USA) starting from 500 ng of total RNA, according to the manufacturer’s instructions. Reverse transcription thermocycling parameters were 65 °C for 5 min, to allow denaturation and annealing, followed by 42 °C for 15 min and 95 °C for 5 min. Glyceraldehyde 3 phosphate dehydrogenase (GAPDH) was amplified as a control. For qPCRs of miRNAs, miRNAs were converted to cDNA with the Universal cDNA synthesis kit II (Exiqon, Vedbaek, Denmark) starting from 500 ng of total RNA. Briefly, RNA was polyadenylated and each miRNA was detected by the mature DNA sequence as the forward primer and a 3′ universal reverse primer provided in the Universal cDNA synthesis kit. Reverse transcription thermocycling parameters were 42 °C for 60 min and 95 °C for 5 min. Human small nuclear U1A1 RNA was amplified as a control. 

### 4.8. qPCR

Prior to qPCR reactions being performed, the cDNA was diluted 1 in 5 and 1 in 80 for the Takara and Exiqon assays, respectively. All qPCR was performed using the SYBR Green master mix SYBR Premix Ex Taq II (Tli RNase H Plus) (Takara Bio, USA, Inc.) in a 10 µL final volume. ′Primer sequences used for miRNA amplification are not currently provided by the kit providers. The primer sequences for the RAF1 amplification were (5′-GAGGAGGAGCGGGCGAGAAG-3′) for the forward primer and (5’- GGAGCCATCAAACACGGCATCT-3′) for the reverse primer. The primer sequences for the ULK1 amplification were (5′-CCTCGCCAAGTCTCAGACGC-3′) for the forward primer and (5′-CCCCACCGTTGCAGTACTCC-3′) for the reverse primer. The primer sequences for the GAPDH amplification were (5′-CAACGACCACTTTGTCAAGCT-3′) for the forward primer and (5′-CTCTCTTCCTCTTGTGCTCTTGC-3′) for the reverse primer. All qPCR performed was conducted at 95 °C for 30 s, and then 40 cycles of 95 °C for 5 s and 60 °C for 30 s. The specificity of the reaction was verified by melt curve analysis. 

### 4.9. miRNA Mimics and siRNA Transfection

SiRNA and miRNA mimic transfections were performed by electroporation using Gene Pulser Xcell Electroporation Systems (Biorad, Hercules, CA, USA). Briefly 5 × 10^6^ cells were electroporated at 950 μF to 250 V in 400 μL IMDM or RPMI medium with 100 nM ALK siRNA, or 100 nM RAF1 siRNA, or miR-7 mimic of a 100-μmol/L stock solution or by the same quantity of a negative control miRNA (miR-Neg) or scrambled siRNA (siSCR) (Eurogentec, Seraing, Liège, Belgium). The siRNA sequences used were (5′-GGGCGAGCUACUAUAGAAATT–3′) for ALK and (5′-GGAACUGUUUAUAAGGGUATT–3′) for RAF1. Following the shock, the cells were rapidly resuspended in 5 mL of IMDM or RPMI supplemented with respectively 20% or 10% FCS. They were subsequently used either for protein extractions, flow cytometry, or for viability/apoptosis assays.

### 4.10. Viability Assays 

Karpas-299 and SU-DHL-1 cells were transfected with miR-7-5p mimic (miR-7) or negative control (miR-Neg) for 24 h, then counted and treated with increasing concentrations of crizotinib (0 to 500 nM for Karpas-299 and 0 to 400 nM for SU-DHL-1). The cells were seeded in 96-well plates (10,000 cells/well, in respectively 100 µL RPMI/10%FBS or IMDM/20% FBS), incubated at 37 °C for an additional 48 h, and the cell viability was assessed using the CellTiter 96 AQueous One Solution cell proliferation assay (Promega, Fitchburg, WI, USA). Additionally, Karpas-299 were counted and seeded in 96-well plates (10,000 cells/well, in 100 μL IMDM/20% FBS). The cells were incubated at 37 °C in the presence of crizotinib 500 nM (Crizo) or vemurafenib 20 µM (VEM), alone or in combination and the cell viability was assessed as stated after 48 h.

### 4.11. Apoptosis Measurement

The analysis of apoptosis was done by annexin V (AnnexinV-FITC, BD Biosciences #556419, Le Pont de Claix, France) and cell viability probe (Via-Probe, BD Biosciences #555815, Le Pont de Claix, France) staining according to standard protocols, followed by flow cytometry using a MACSQuant VYB cytometer from Miltenyi Biotec (Paris, France) and FlowJo software was used for results analysis.

### 4.12. Western-Blotting 

The cells were lysed in radioimmunoprecipitation assay (RIPA) buffer (Tris HCl, 20 mM, pH 7.4, NaCl 150 mM, EDTA 4 mM, Triton X-100 1%, and SDS 0.2%) supplemented with phosphatase inhibitors (Na_3_VO_4_ 1 mM, NaF 1 mM) and phenylmethylsulfonylfluoride (PMSF) 1 mM, purchased from Sigma-Aldrich, and protease inhibitor cocktail (Roche, Penzberg, Germany). The protein lysates were fractionated on SDS-PAGE (10% or 15%), and transferred to a nitrocellulose membrane (Whatman) (GE Healthcare, Little Chalfont, UK). Western blotting was performed using ALK (D5F3 XP, Cell Signaling Technology #3633, Ozyme, Saint Cyr l’Ecole, France), phospho-ALK (Y1507) (Cell Signaling Technology, #14678), LC3-B (Cell Signaling Technology #2775), p62 (BD Transduction Laboratories #610832), ULK1 (Cell Signaling Technology #6439), phospho-ULK1 (Ser757) (Cell Signaling Technology #14202), RAF1 (Cell Signaling Technology #3738), MEK1/2 (Cell Signaling Technology #9122), phospho-MEK1/2 (Ser217/221) (Cell Signaling Technology #9121), ERK1/2 (Cell Signaling Technology #9102), phospho-ERK1/2 (Thr202/Tyr204) (Cell Signaling Technology #9106), β-actin (Santa Cruz #7210, Heidelberg, Germany), and HSP90 (Origene #TA332385, Rockville, MD, USA) antibodies. The proteins were visualized using the Clarity™ ECL Western Blotting Substrate (Bio-Rad, Hercules, CA, USA).

### 4.13. Measure of the Autophagic Flux by Flow Cytometry

The RFP-GFP-LC3 cells were harvested, washed in PBS, and resuspended in 1 mL of PBS. To assess the autophagic flux, the fluorescence ratio of RFP/EGFP was measured using a BD LSRFortessa™ X-20 flow cytometer. As stated by Kimura et al., the use of this RFP-GFP reporter system for autophagic flux quantification takes advantage of the higher sensitivity of EGFP fluorescence to the acidic environment of the autolysosome relative to RFP [99]. Thus, cells with high autophagic flux become less green (because of the fusion of the autophagosomes with lysosomes), and harbor an increased RFP/EGFP ratio. The results analysis was performed using the FlowJo software.

### 4.14. Immunofluorescence Staining

Cells were seeded onto glass slides (Fisher Scientific) coated with 0.01% poly-L-lysine (Sigma), then fixed in 4%formaldehyde for 8 min. After phosphate buffered saline (PBS) washes, the cells were incubated in 0.01% saponin containing 3% BSA for 30 min and then incubated with anti-LC3B antibodies (MBL, PM036, 1/700) for 45 min. The cells were then washed before incubation with an anti-rabbit Alexa-488 secondary antibody (Invitrogen, 1/800) for 30 min, followed by PBS and distilled water washes and mounting in ProLongTMGold antifade medium with DAPI (4′6-diamidino-2-phenylindole, Invitrogen). Images were acquired using a confocal Zeiss LSM 780. For quantification, fields were chosen arbitrarily based on DAPI staining, and the number of LC3B dots per cell of at least 100 cells per independent experiment was determined with Image J software (U.S. NIH, Bethesda, MD, USA).

### 4.15. RNA Pulldown Using Biotinylated miRNA

Karpas-299 cells were harvested 18 h post-transfection with biotinylated miR-7-5p (Exiqon, Vedbaek, Denmark) or biotinylated cel-miR-39 (negative control, Exiqon) in IP-buffer composed of 25 nM Tris-HCl pH 7.5, 200 nM NaCl, 0.2% Triton-X100, 5 mM MgAc, 1 mM DTT, protease inhibitors (Roche, Penzberg, Germany) and 0.2 U/µL RNase OUT (Invitrogen, Carlsbad, CA, USA). Magnetic beads coupled with streptavidin were first incubated with BSA (0.5 mg/mL) and yeast tRNA (0.2 mg/mL) for 30 min at 4 °C and then washed twice with the IP buffer. The cells were then lysed by sonication, and the lysates were incubated with the streptavidin beads for 2 h at 4 °C. The beads were then washed five times with 1 mL of IP buffer. The remaining RNAs were then extracted using the Trizol reagent (Invitrogen, Carlsbad, CA, USA), according to the manufacturer’s instructions. The biotin pulldown efficiency was expressed as a percentage of the input. 

### 4.16. In Vitro Kinase Assay

The recombinant full-length human ULK1 (H00008408-P01) and recombinant human active RAF1 protein (4540-KS) were purchased from Bio-Techne (Rennes, France). The in vitro kinase assay was performed by incubating ULK1 (200 ng) and RAF1 (20 ng) at 30 °C for 1 h in reaction buffer (25 mM MOPS pH 7.2, 25 mM MgCl_2_, 2 mM EDTA, 5 mM EGTA, and 0.25 mM DTT) with or without vemurafenib 100 nM (RAF1 inhibitor) and 200 µM ATP. The kinase reaction was stopped by the addition of Laemmli buffer 2×, and the samples were then heated for 5 min at 95 °C, loaded onto SDS-PAGE, transferred to a nitrocellulose membrane, and probed with the RAF1 (Cell Signaling Technology #3738), total ULK1 (Cell Signaling Technology #6439), and p-ULK1 (Ser 757) (Cell Signaling Technology #14202) antibodies.

### 4.17. Statistical Analysis

The results are presented as the mean values ± standard deviations (SD) from at least three independent experiments unless otherwise indicated. Determination of the statistical significance was performed using an unpaired Student *t*-test for side by side comparison of two conditions. Welsch’s correction was applied when the variances were significantly different.

## 5. Conclusions

NPM-ALK+ ALCL treatment still requires improvement to prevent refractory/relapse cases following standard chemo- or NPM-ALK targeted-therapies. Our study provides evidence, for the first time, that the dual inhibition of NPM-ALK and RAF1 (using pharmacological (vemurafenib) or molecular approaches (siRNA or miRNA (miR-7-5p mimics) targeting RAF1)) may be superior to single NPM-ALK targeted therapies (crizotinib) in killing tumor cells. We found that this combined therapy triggered enhanced autophagy, notably through the relieved inhibition of the ULK1 protein, and ultimately led to lymphoma cell death. Thus, our work stresses the importance of autophagy in the responses to anti-cancer drugs and highlights a new therapeutic approach for NPM-ALK+ ALCL.

## Figures and Tables

**Table 1:** miR-7-5p was down-regulated upon crizotinib treatment in NPM-ALK+ Karpas-299 cells. A set of 10 selected miRNAs under-expressed or over-expressed with the highest *p*-values in crizotinib-treated (500 nM, 24 h) versus untreated NPM-ALK+ Karpas-299 cells is presented.

**Genes Under-Expressed in Crizotinib Treated Versus Untreated Cells**		
**Name**	**Fold Regulation**	***p*-Value**
hsa-miR-7-5p	−2.6411	0.000594
hsa-miR-222-5p	−6.7695	0.001684
hsa-miR-221-5p	−2.6084	0.001993
hsa-miR-19a-5p	−4.402	0.01234
hsa-miR-92a-1-5p	−2.7998	0.017765
hsa-miR-885-5p	−2.2267	0.021152
hsa-miR-146a-5p	−2.0383	0.035913
hsa-miR-363-5p	−2.8992	0.043604
hsa-miR-143-3p	−2.0704	0.047823
hsa-miR-10b-3p	−5.0548	0.049052
**Genes Over-Expressed in Crizotinib Treated Versus Untreated Cells**		
**Name**	**Fold Regulation**	***p*-Value**
hsa-miR-15a-5p	2.0232	0.000008
hsa-miR-425-5p	2.4347	0.000077
hsa-miR-9-5p	2.1883	0.000089
hsa-miR-219a-5p	3.0043	0.000116
hsa-miR-31-5p	3.2204	0.000226
hsa-miR-26a-5p	2.2241	0.000308
hsa-miR-425-3p	2.1882	0.000335
hsa-miR-191-5p	2.0509	0.001091
hsa-miR-497-5p	6.1522	0.002711
hsa-miR-190a-5p	2.1526	0.01914

## Supplementary Materials

The following are available online at https://www.mdpi.com/2072-6694/12/10/2951/s1, Supplementary file 1, Figure S1: miR-7-5p mimics increase basal and crizotinib-induced autophagy flux. Figure S2: *RAF1* mRNA is a target of miR-7-5p; Figure S3: NPM-ALK molecular or pharmacological inactivation increases RAF1 mRNA and protein levels; Figure S4: Status of MEK and phospho-MEK upon ALK and RAF1 inhibition; Figure S5: Effects of miR-7-5p mimics and vemurafenib, as single or combined treatment, on cell viability. **Figure S6**: Vemurafenib increases the basal and crizotinib-induced autophagy flux. Figure S7: RAF1 molecular inactivation potentiates crizotinib-induced apoptosis; Figure S8: Effect of combined ALK and RAF inhibition on ULK1 Serine 757 phosphorylation. Supplementary file 2, Uncropped western blot figures.
