# Supplementary material for: High Levels of miR-7-5p Potentiate Crizotinib-Induced Cytokilling and Autophagic Flux by Targeting RAF1 in NPM-ALK Positive Lymphoma Cells
Source: Cancers (Basel). 2020 Oct 13;12(10):2951. doi: 10.3390/cancers12102951 (PMC7650725; doi:10.3390/cancers12102951)
Supplement: Supplementary file 1 [file cancers-12-02951-s001.zip › cancers-967315-Supplementary Material File 1.pdf]

Supplementary Materials: High Levels of miR-7-5p Potentiate Crizotinib-Induced Cytokilling and Autophagic Flux by Targeting RAF1 in NPM-ALK Positive Lymphoma Cells

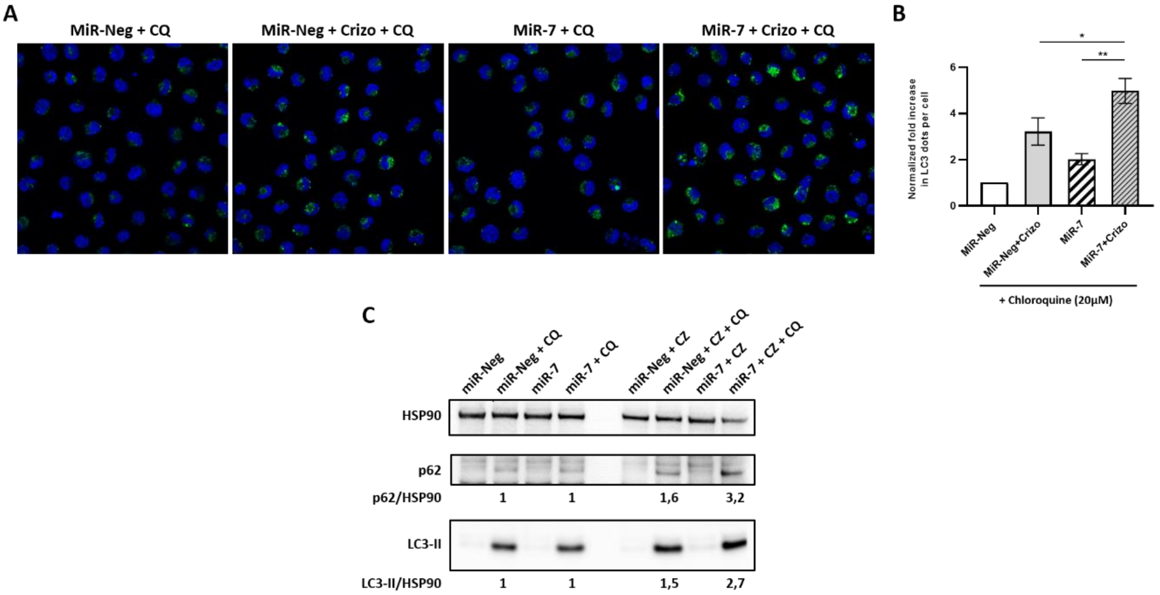

Figure S1: miR-7-5p mimics increase basal and crizotinib-induced autophagy flux. Karpas-299 cells transfected for 72h with either scramble miRNA (miR-Neg) or miR-7-5p mimics (miR-7), were treated or not for the last 24h with crizotinib (Crizo) (250nM), in the presence of chloroquine (CQ, 20µM), before staining for LC3B. Representative confocal pictures are shown in **(A)**, and the graph in **(B)** represents the normalized fold increase in the number of LC3B dots per cell, in comparison with the control condition, assigned to 1. Data represent mean ± SD; n=3; unpaired Student's t test. **(C)** Representative western-blot showing LC3 and p62 protein levels in Karpas-299, treated as in (A). HSP90 was used as a loading control. LC3-II/HSP90 and p62/HSP90 densitometric ratios are indicated.

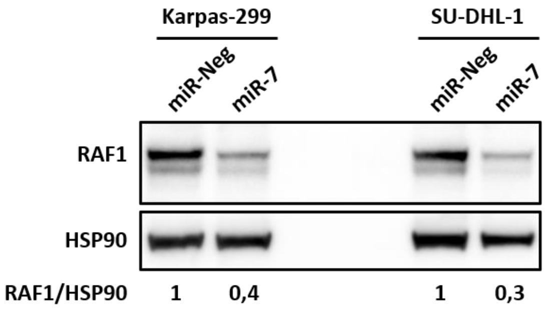

Figure S2: RAF1 mRNA is a target of miR-7-5p. Representative western-blot showing RAF1 protein levels 48h following control miRNA (miR-Neg) or miR-7-5p (miR-7) transfection in Karpas-299 and SU-DHL-1 cells. HSP90 was used as a loading control. RAF1/HSP90 densitometric ratios are indicated.

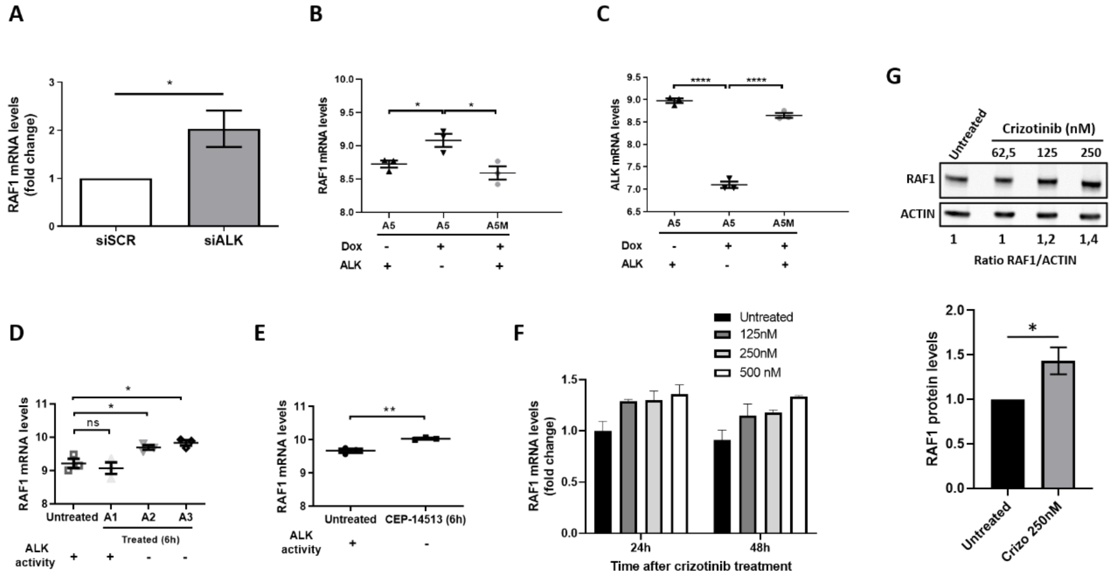

Figure S3: NPM-ALK molecular or pharmacological inactivation increases RAF1 mRNA and protein levels. (A) Karpas-299 cells were transfected for 72h with ALK targeted siRNA (siALK) or scramble siRNA (siSCR) and analysed by qRT-PCR for *RAF1* mRNA levels. Results are expressed as fold change in *RAF1* mRNA levels in siALK transfected cells, in comparison with siSCR conditions, assigned to 1. Data represent mean ± SD; n=3; *p≤0.1; unpaired Student's t test. **(B)** *RAF1* and **(C)** *ALK* mRNA levels were assessed by microarray analysis (GeneChIp Human Genome U133A, Affymetrix) in the TS-TTA-A5 cell line (a Sup-M2-derived human NPM-ALK+ ALCL cell line which expresses a doxycycline (Dox)-inducible shRNA directed against the ALK mRNA), treated (+) or not (-) with Dox (84h). A5: Wild-type ALK shRNA sequence. A5M: mutated ALK shRNA sequence. (Piva et al, 2006 - publicly available data) [63]. **(D)** *RAF1* mRNA levels were assessed as described in (B) in TS-TTA-A5 cells that were treated for 6h with the cell-permeable pyrrolocarbazole-derived ALK inhibitors : A1 for mock-compound (CEP-11988), A2 for CEP-14083 and A3 for CEP-14513 drugs (Piva et al, 2006; publicly available data) [63] and **(E)** in the SU-DHL-1 cells, treated with the CEP-14513 ALK inhibitor (Marzek et al, 2013; publicly available data) [64]. **(F)** Karpas-299 cells were treated or not for 24h and 48h with crizotinib at 125, 250 and 500nM. *RAF1* mRNA levels were analysed by qRT-PCR. Results are expressed as normalized fold change in *RAF1* mRNA levels in crizotinib-treated cells, in comparison with the untreated condition at 24h, assigned to 1. Data represent mean ± SD; n=2. **(G)** Upper part: Karpas-299 cells were treated or not (Untreated) for 24h with increasing doses of crizotinib as indicated, and assessed by western-blotting for RAF1 protein levels. Actin was used as a loading control. RAF1/Actin densitometric ratios are indicated. Lower part: Histogram representation of the RAF1 protein levels in Karpas-299 cells treated or not (Untreated) for 24h with crizotinib (Crizo) (250 nM). Data represent mean ± SD; n=3; *p≤0.1; unpaired Student's t test.

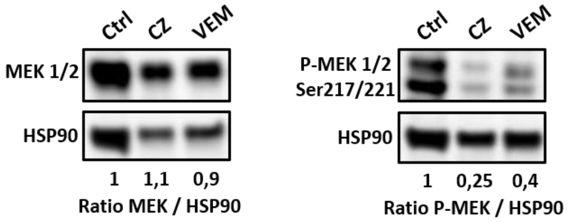

Figure S4: Status of MEK and phospho-MEK upon ALK and RAF1 inhibition. Karpas-299 cells were treated or not (Ctrl) overnight (16h) with crizotinib (CZ) 250nM or vemurafenib (Vem) 10µM. The efficiency of the vemurafenib treatment was assessed by western-blotting for decreased phospho-MEK. Whole cell lysates were loaded twice on a same gel and probed for total MEK and phospho-MEK contents. MEK/HSP90 and P-MEK/HSP90 densitometric ratios are indicated.

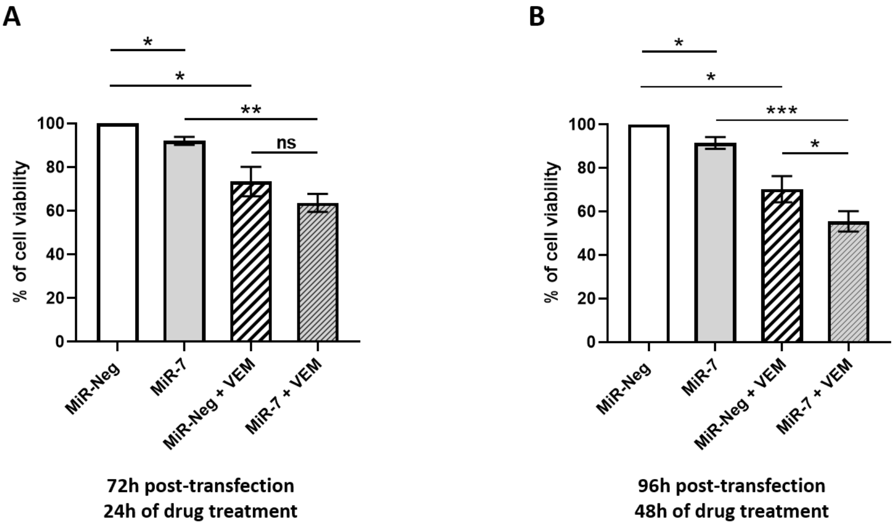

Figure S5: Effects of miR-7-5p mimics and vemurafenib, as single or combined treatment, on cell viability. Karpas-299 cells transfected for 72h or 96h with either scramble miRNA (miR-Neg) or miRNA-7-5p mimics (miR-7), and treated or not for the last 24h (panel A) or 48h (panel B), respectively, with vemurafenib (VEM) (20µM). Cell viability was determined by MTS assay. The graphs represent mean values ± SD from three independent experiments. Data represent mean ± SD; n=3; ns: not significant; *p≤0.1; **p ≤ 0.01; ***p ≤ 0.001; unpaired Student's t test.

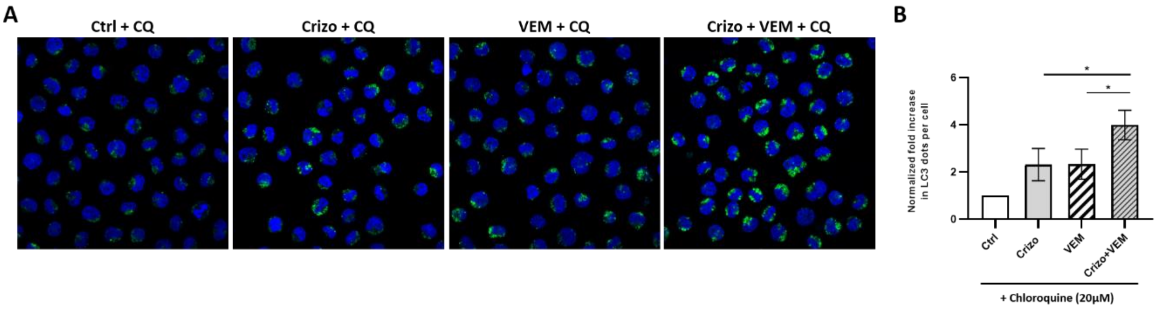

Figure S6: Vemurafenib increases the basal and crizotinib-induced autophagy flux. Karpas-299 cells were treated or not (Ctrl) for 24h with crizotinib (Crizo, 250nM) or vemurafenib (VEM, 20µM), alone or in combinaison, in the presence of chloroquine (20µM), before staining for LC3B. Representative confocal pictures are shown in (A), and the graph in (B) represents the normalized fold increase in the number of LC3B dots per cell, in comparison with the control condition, assigned to 1. Data represent mean ± SD; n=3; unpaired Student's t test.

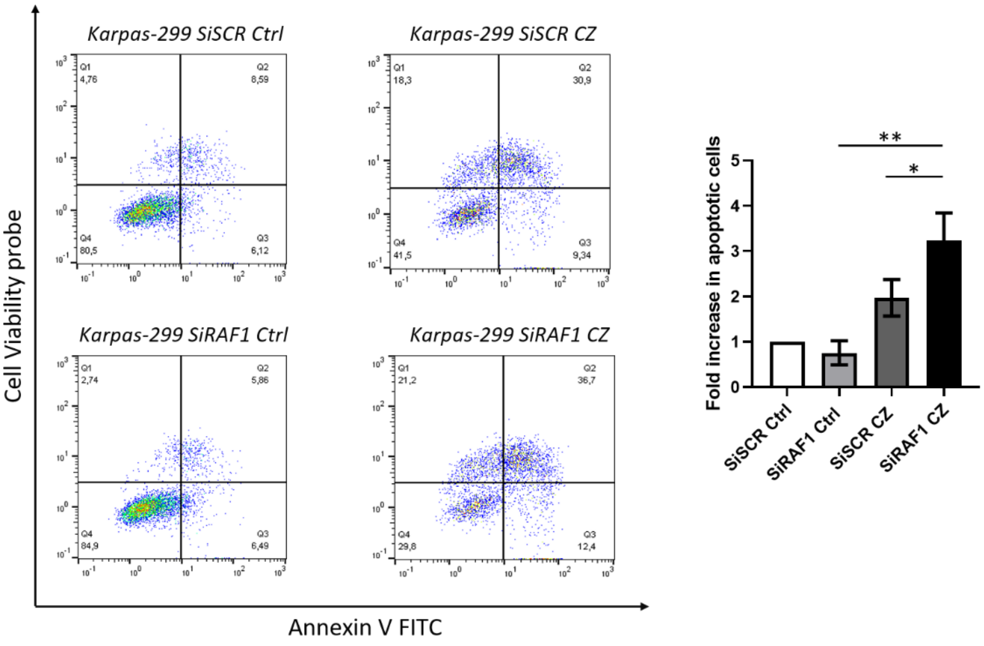

Figure S7: RAF1 molecular inactivation potentiates crizotinib-induced apoptosis. Karpas-299 cells were transfected for 36h with siRNA targeting the *RAF1* mRNA (siRAF1) or corresponding negative controls (siSCR) and treated (CZ) or not (Ctrl) for the last 18h with crizotinib (125nM). Left part: Representative FACS plots of annexin V-positive Karpas-299 cells are shown. Right part: Graph represents fold increase in the percentage of apoptotic cells (including early (AnnexinV positive, Via-Probe negative) and late (AnnexinV positive, Via-Probe positive) apoptosis) in comparison with the siSCR control condition, assigned to 1. Data represent mean ± SD; n=3; *p ≤ 0.1; **p ≤ 0.01; unpaired Student's t test.

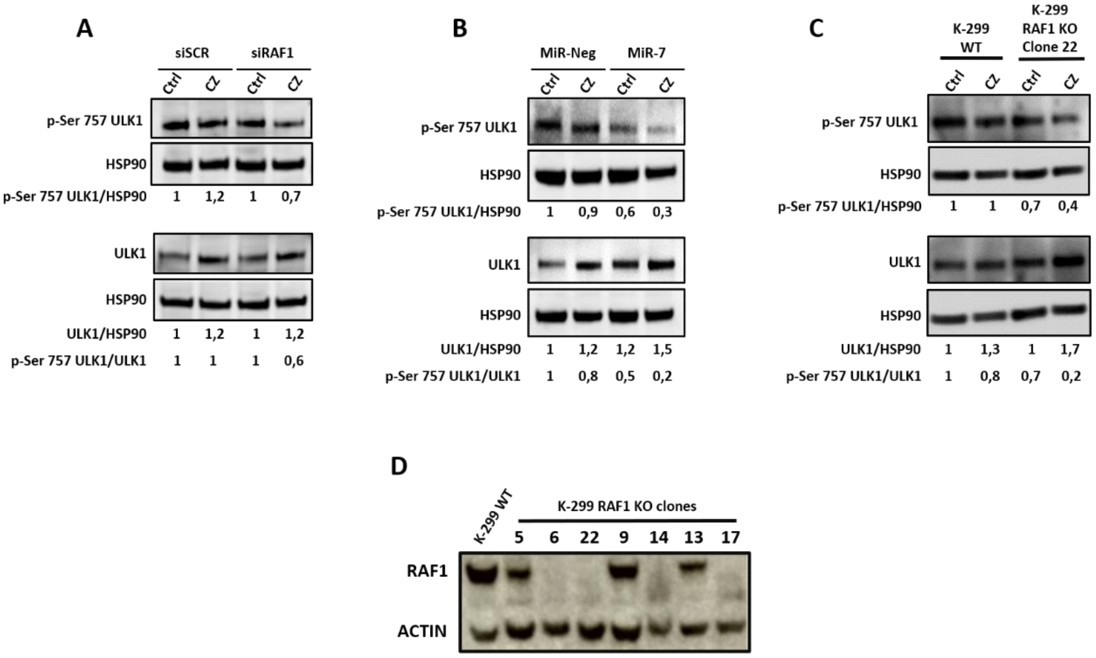

Figure S8: Effect of combined ALK and RAF inhibition on ULK1 Serine 757 phosphorylation. (A) Karpas-299 cells were transfected for 72h either with scramble (siSCR) or RAF1 (siRAF1) targeting siRNAs or **(B)** with scramble microRNA (miR-Neg) or microRNA-7-5p mimics (miR-7). Cells were treated or not (Ctrl) for the last 16h with crizotinib (CZ) 250nM. **(C)** Wild-type Karpas-299 cells (K-299 WT) or Karpas-299 cells knock-downed for RAF1 (K-299 RAF1 KO) using the CRISPR/Cas9 system were treated or not (Ctrl) with crizotinib (CZ) 250nM. For panels A, B and C, whole cell lysates

were loaded twice on a same gel and probed for total ULK1 and phospho-Ser757 ULK1 contents by western blot. Densitometry analysis of the phospho-ULK1 (Serine757) levels was calculated relative to the control samples (normalized over the hsp90 signals) and relative to the total ULK1 signals. The data are representative of two independent experiments. **(D)** Representative western-blot showing RAF1 protein levels in Karpas-299 wild-type cells (K-299 WT) and in puromycin selected and single cell dilution generated clones for RAF1 depletion (K-299 RAF1 KO). ACTIN was used as a loading control.
