# Supplementary material for: High Levels of miR-7-5p Potentiate Crizotinib-Induced Cytokilling and Autophagic Flux by Targeting RAF1 in NPM-ALK Positive Lymphoma Cells
Source: Cancers (Basel). 2020 Oct 13;12(10):2951. doi: 10.3390/cancers12102951 (PMC7650725; doi:10.3390/cancers12102951)

Full blots for Figure 1E

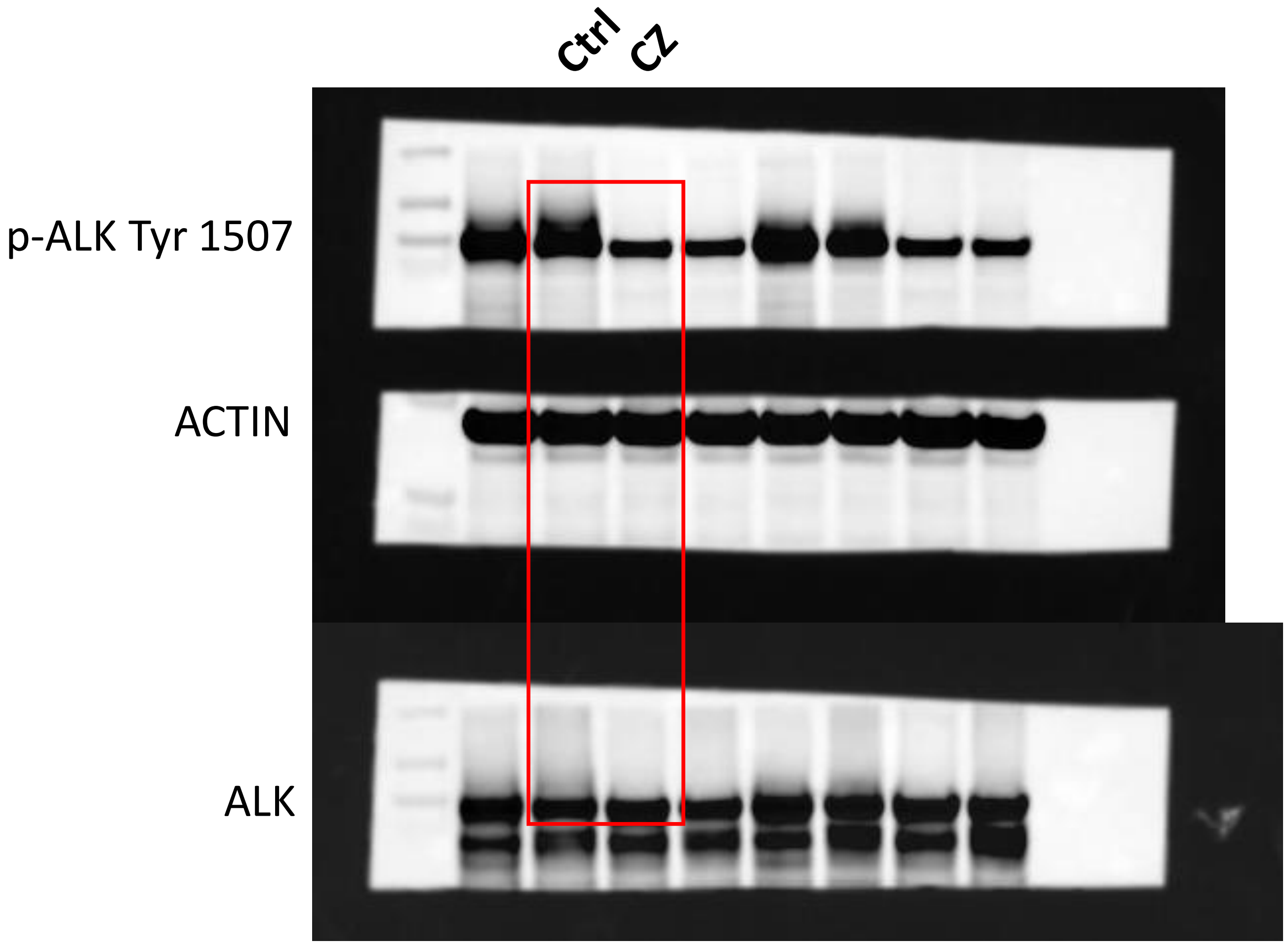

Full blots for Figure 1F

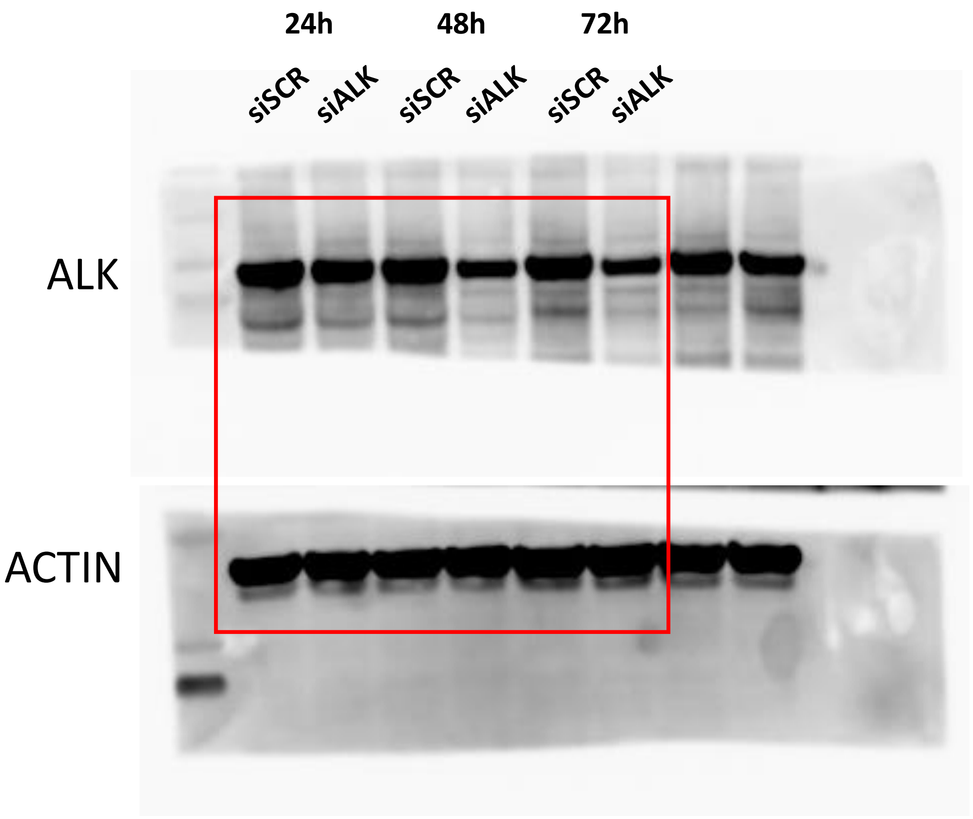

Full blots for Figure 3D

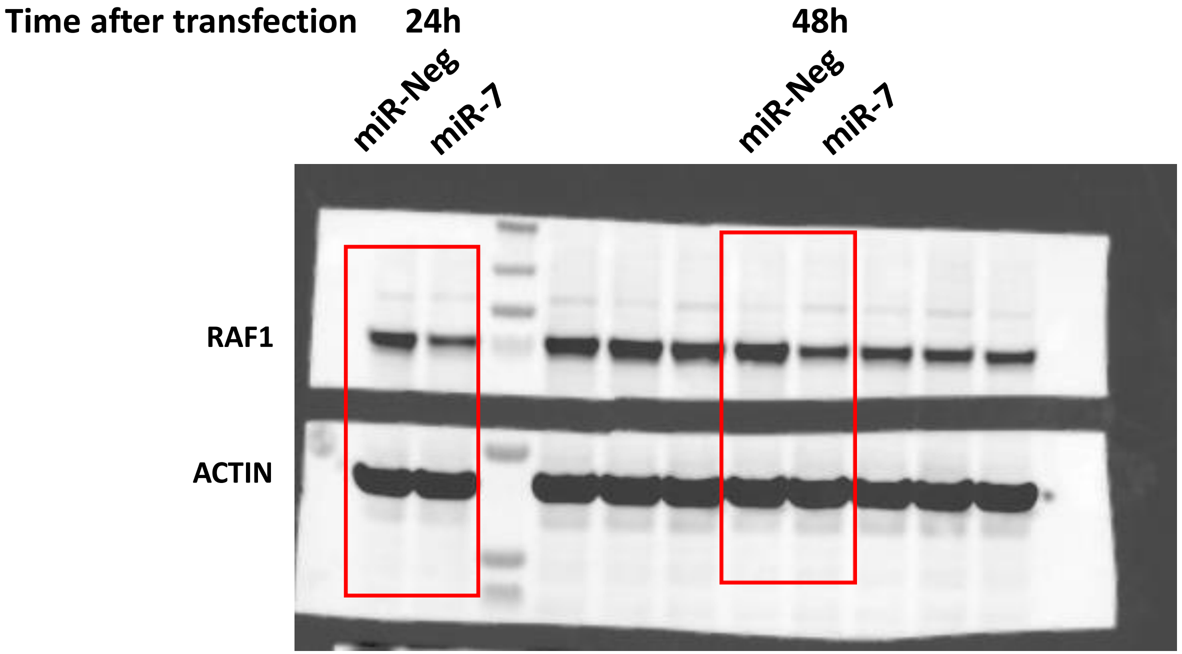

Full blots for Figure 4C

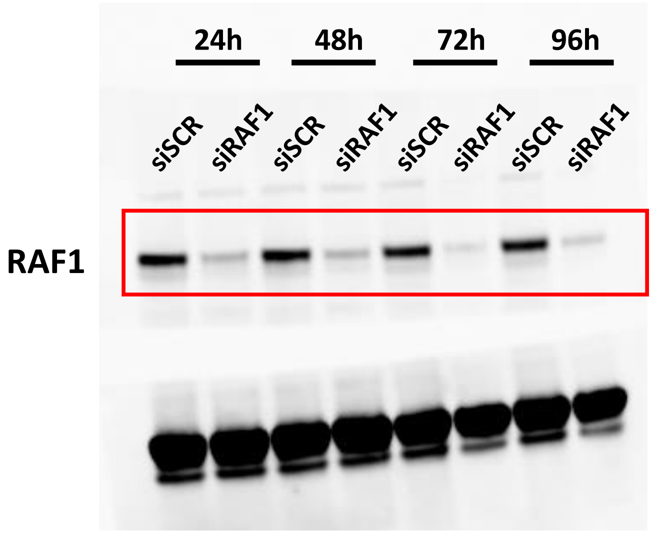

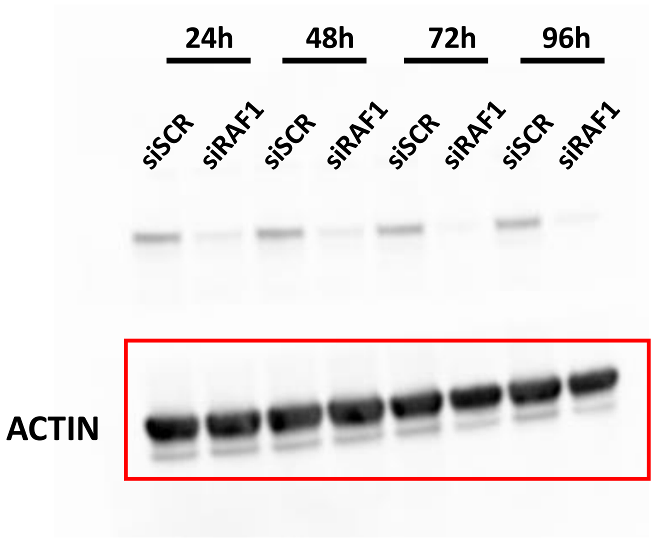

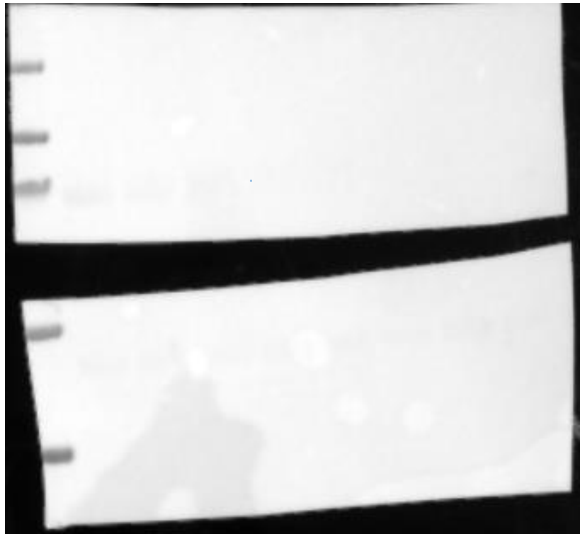

Full blots for Figure 5A

p-Ser 757 ULK1

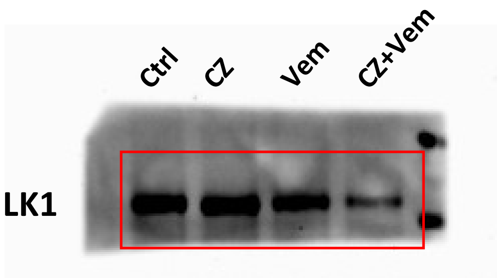

ULK1

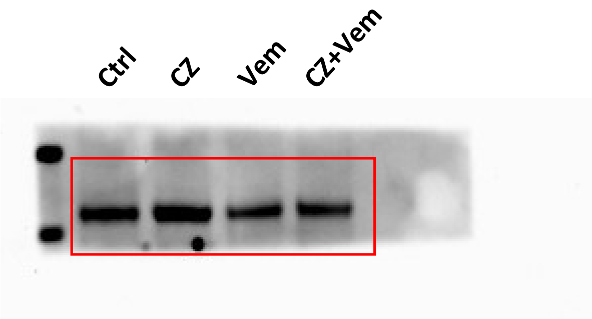

HSP 90

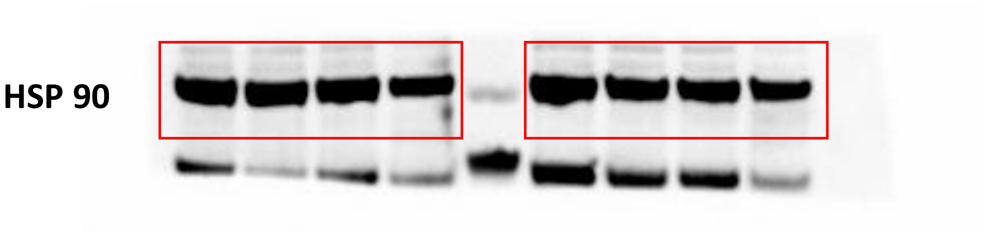

Full blots for Figure 5B

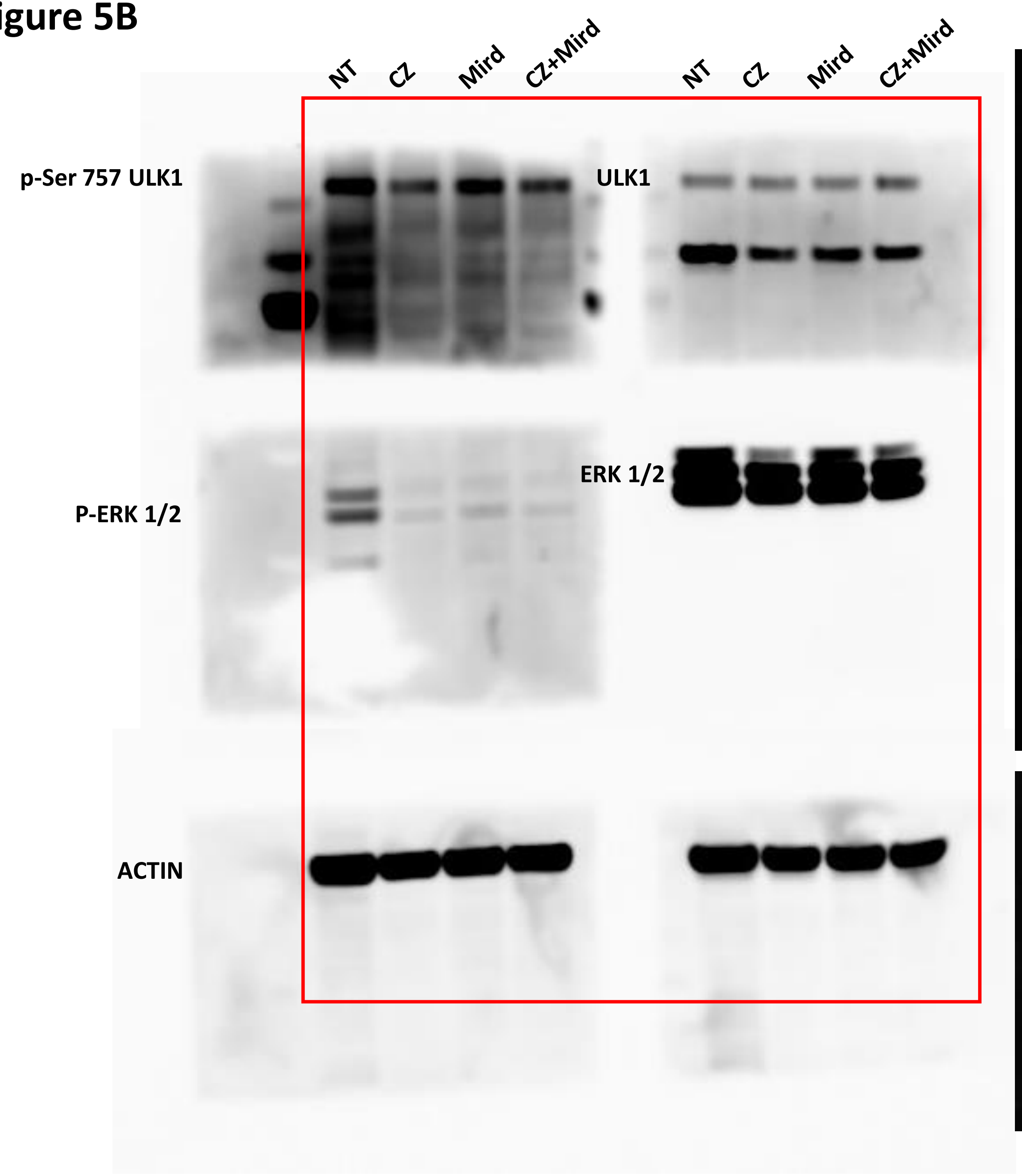

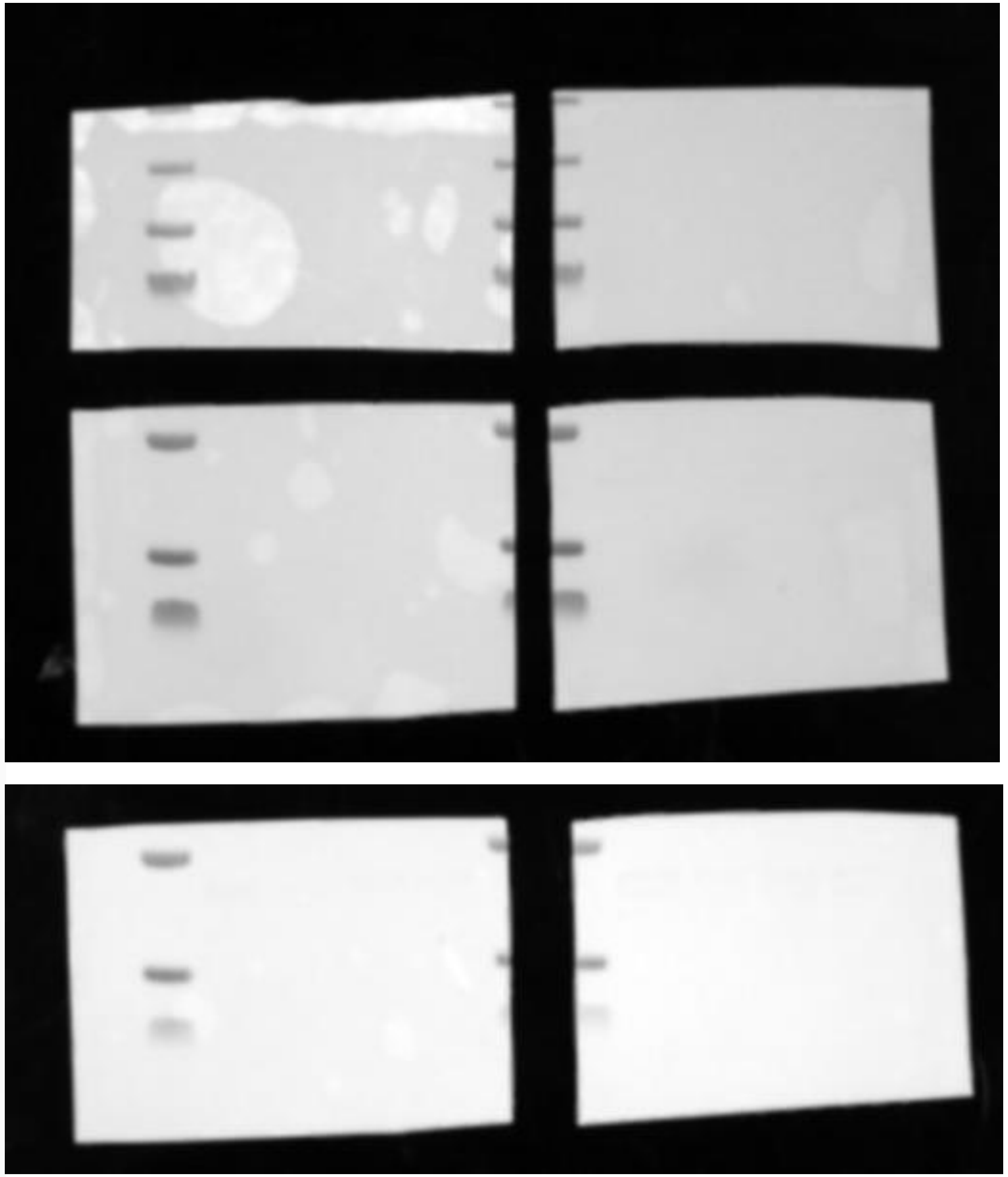

Full blots for Figure 6

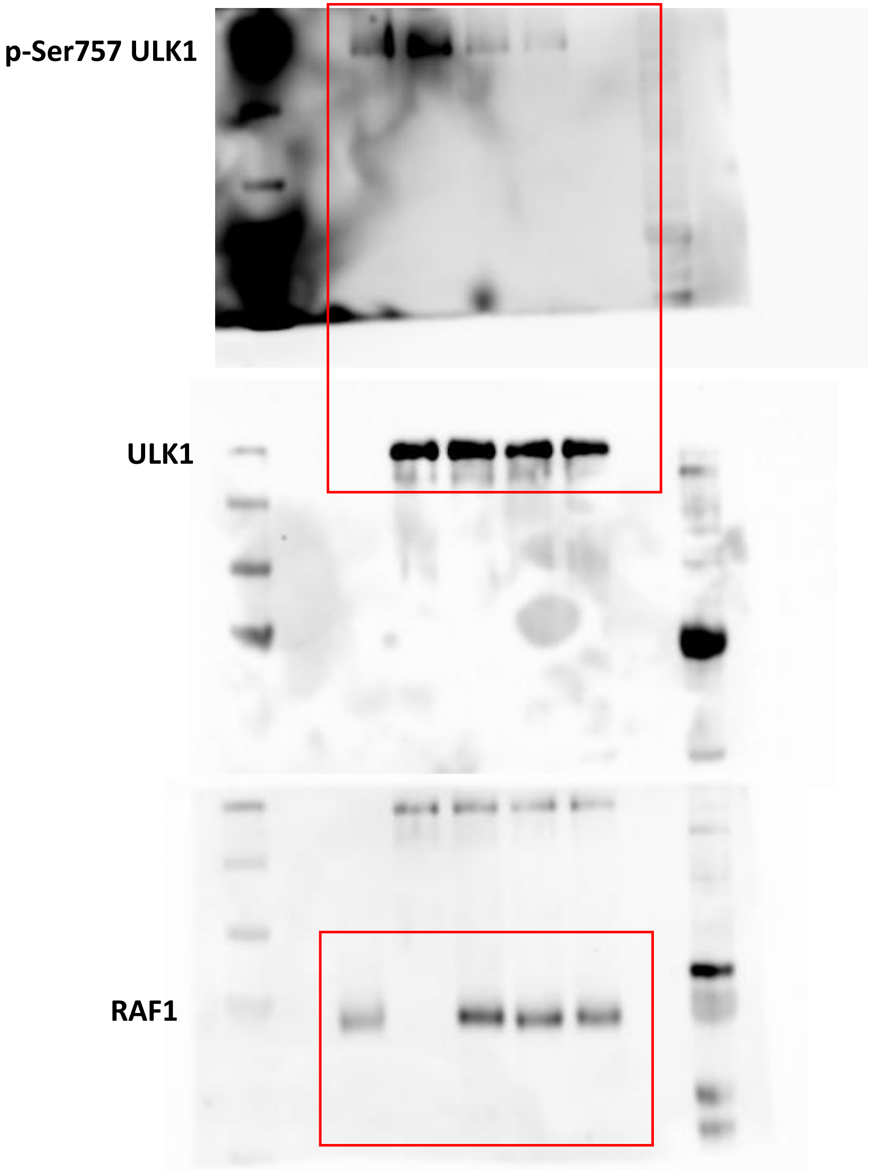

ULK1	-	+	+	+	+
RAF1	+	-	+	+	+
ATP	+	+	+	-	+
Vemurafenib	-	-	-	-	+

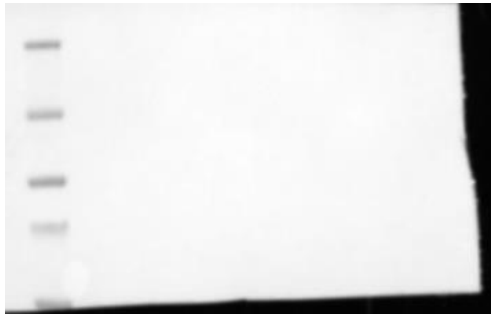

Full blots for Supplemental Figure 1C

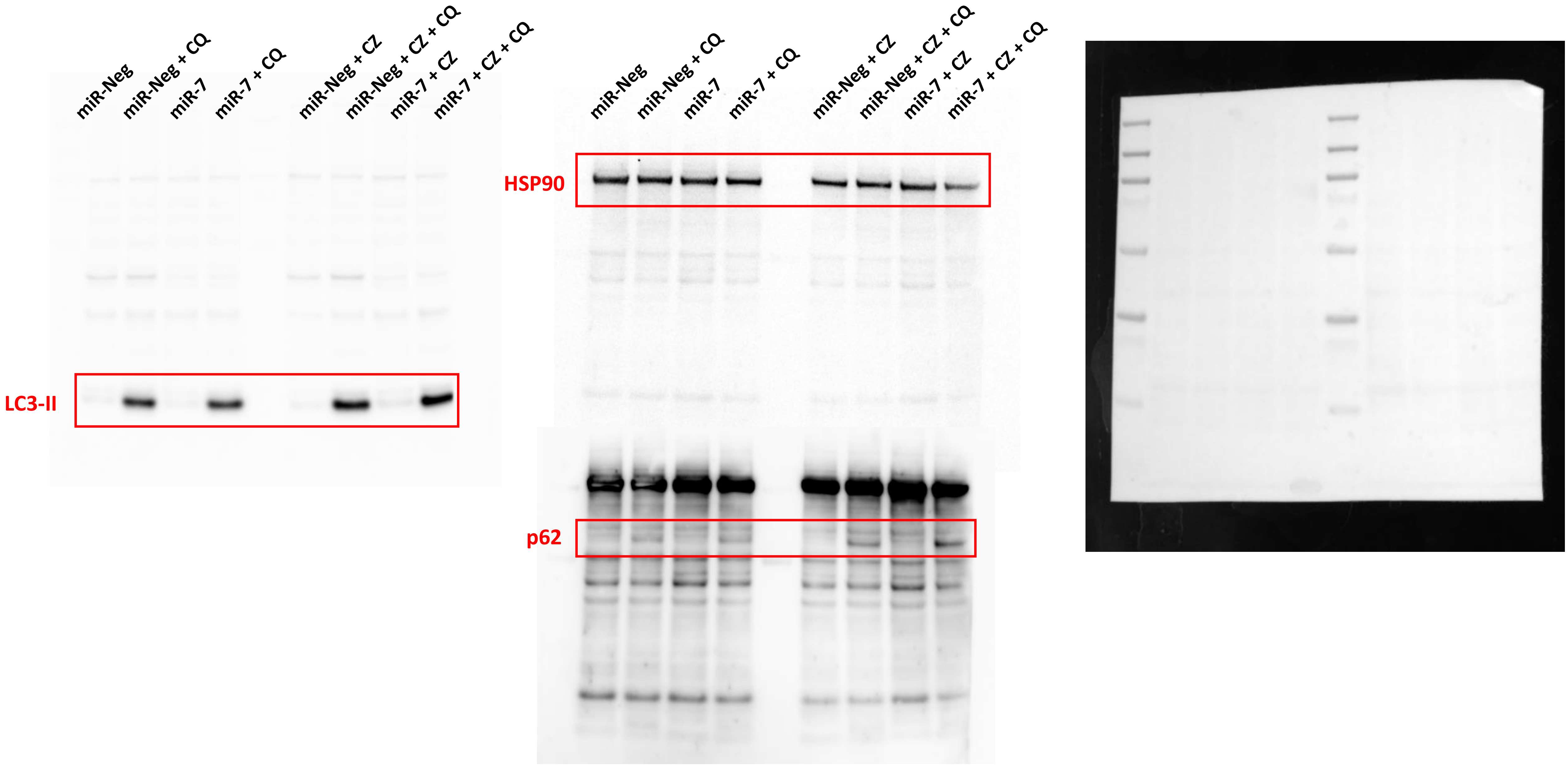

Full blots for Supplemental Figure 2

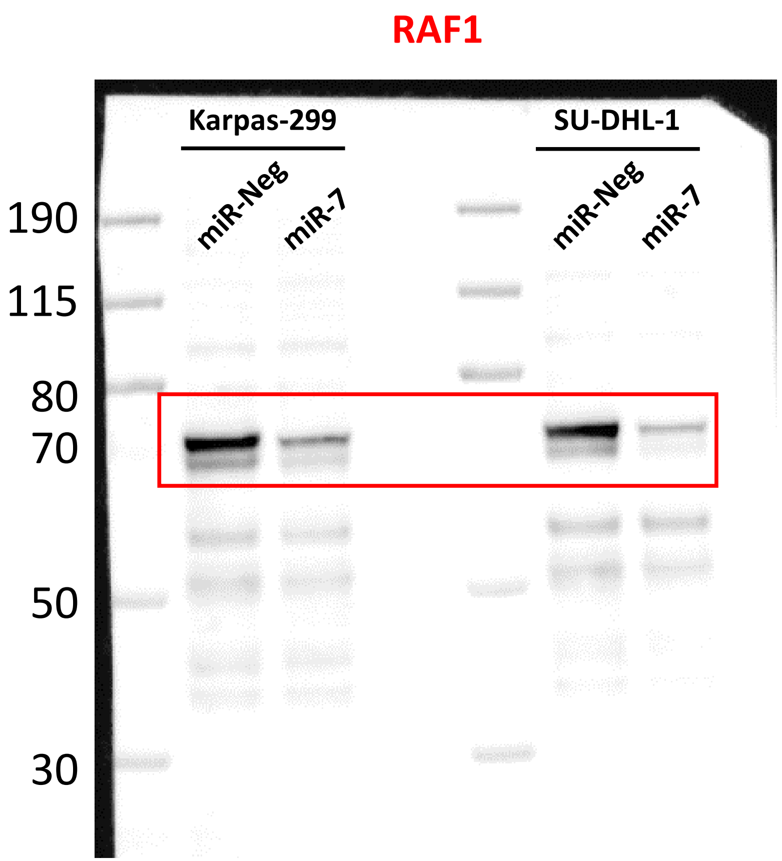

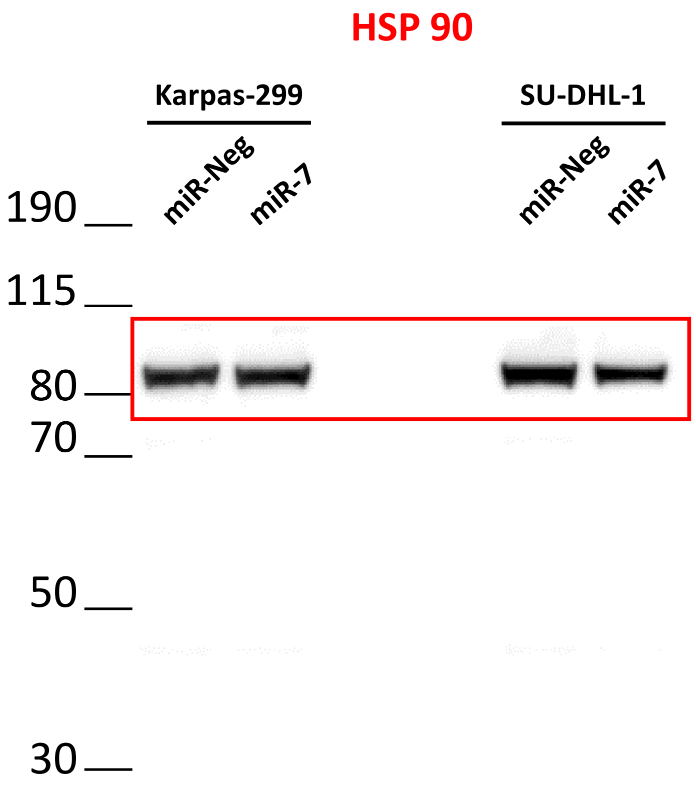

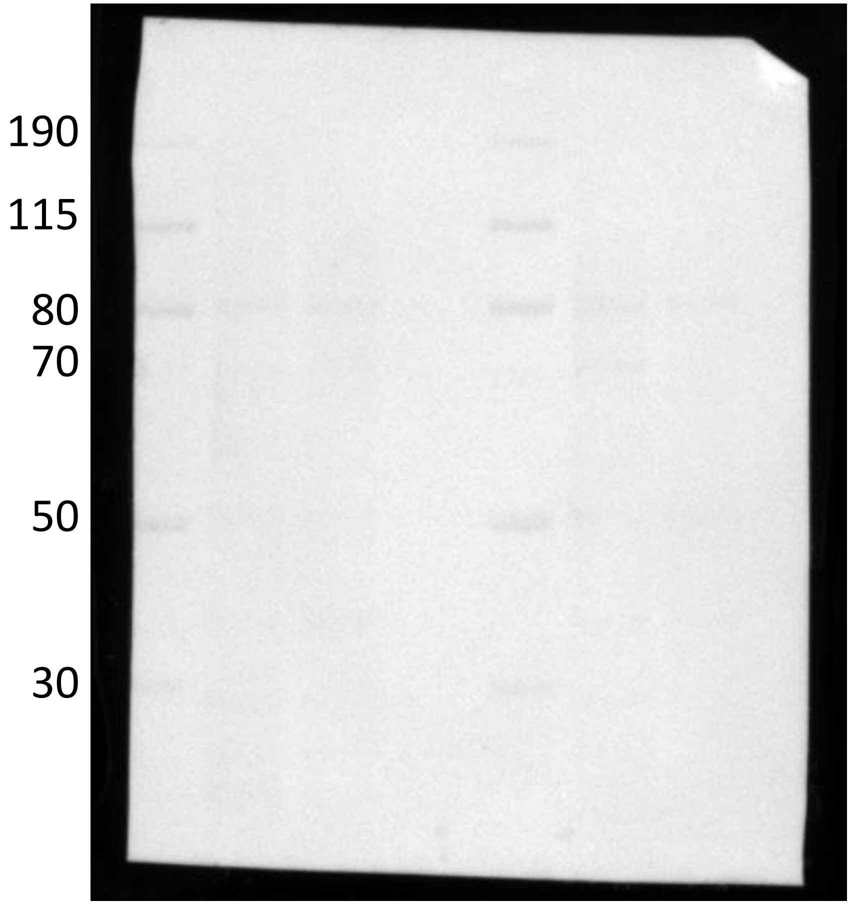

Full blots for Supplemental Figure 3G

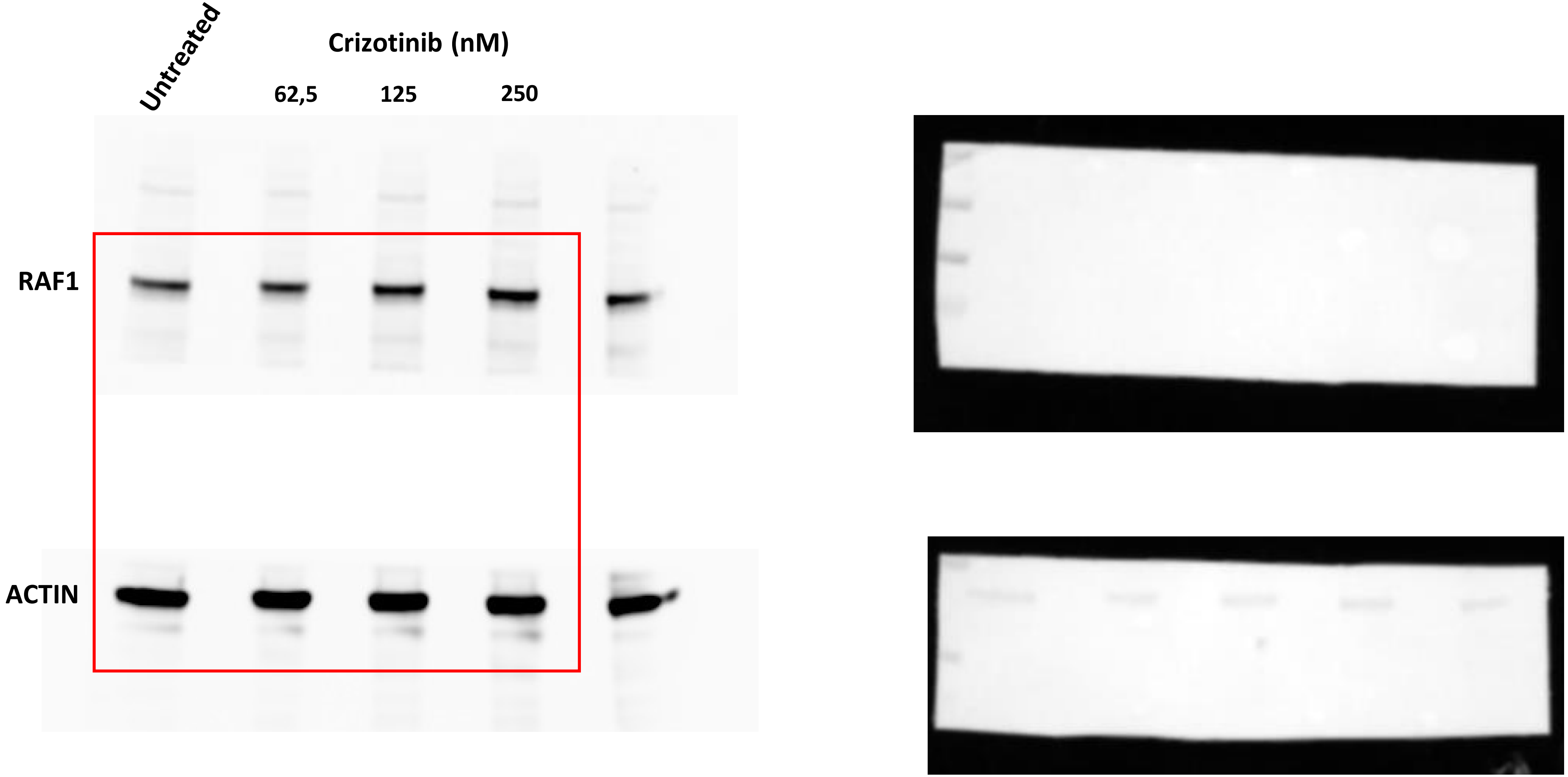

Full blots for Supplemental Figure 4

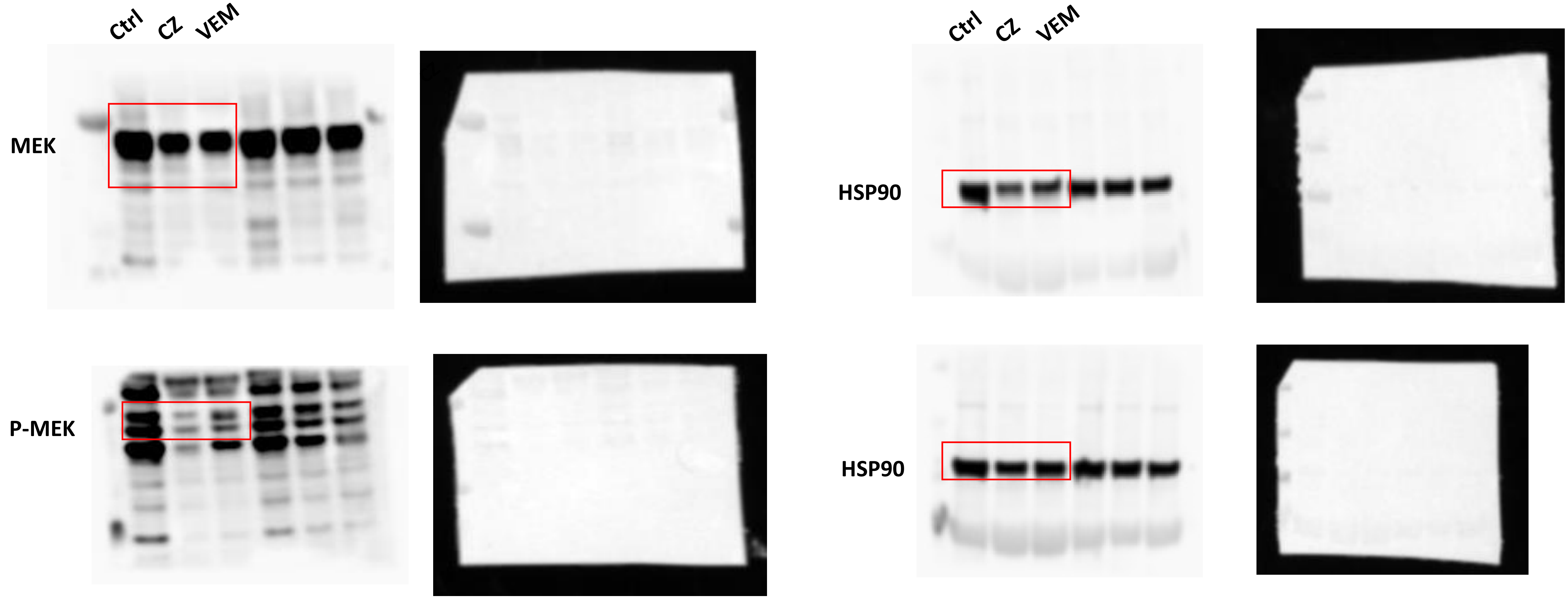

Full blots for Supplemental Figure 8A

p-ULK1 Ser 757

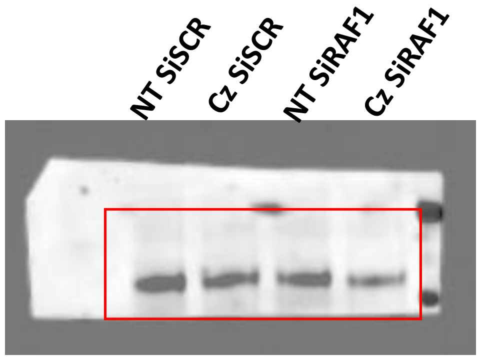

ULK1

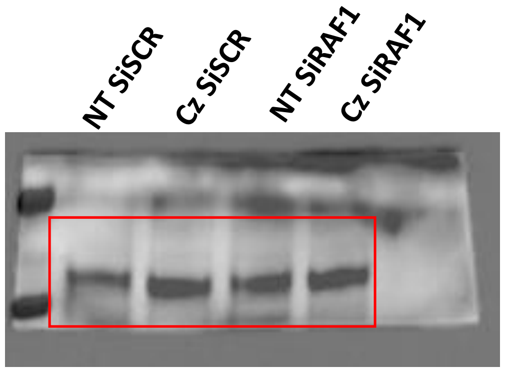

HSP90

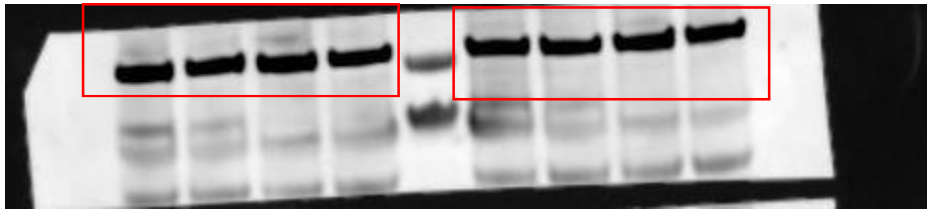

Full blots for Supplemental Figure 8B

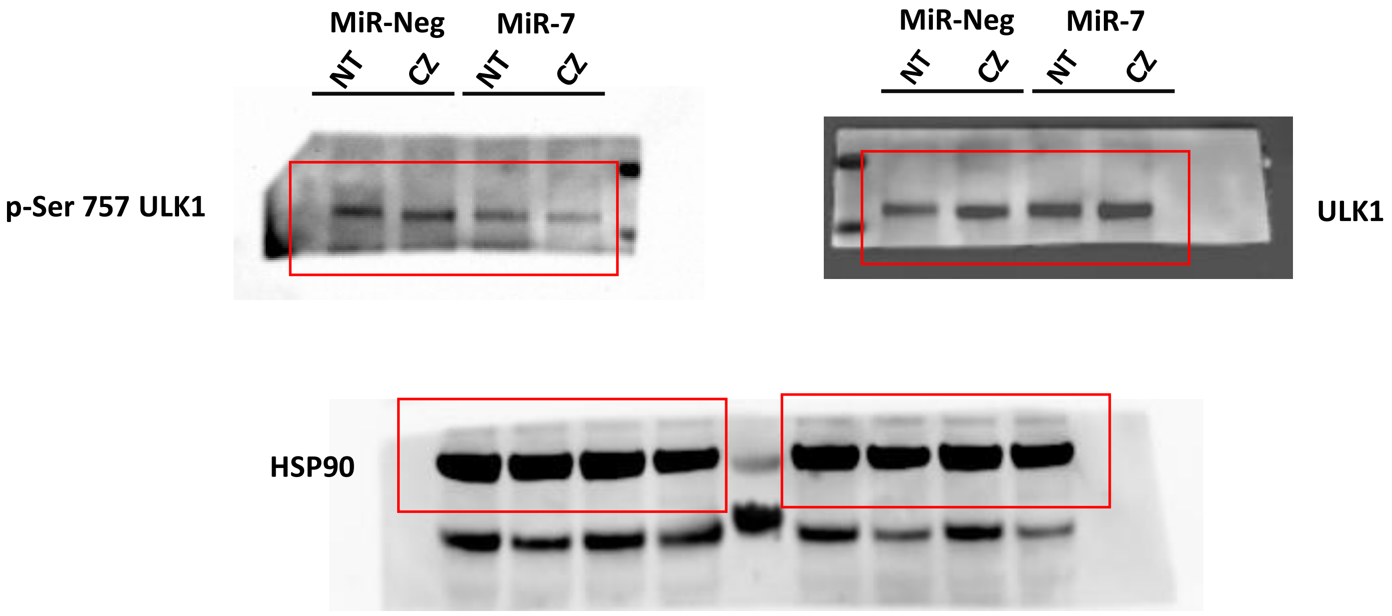

Full blots for Supplemental Figure 8C

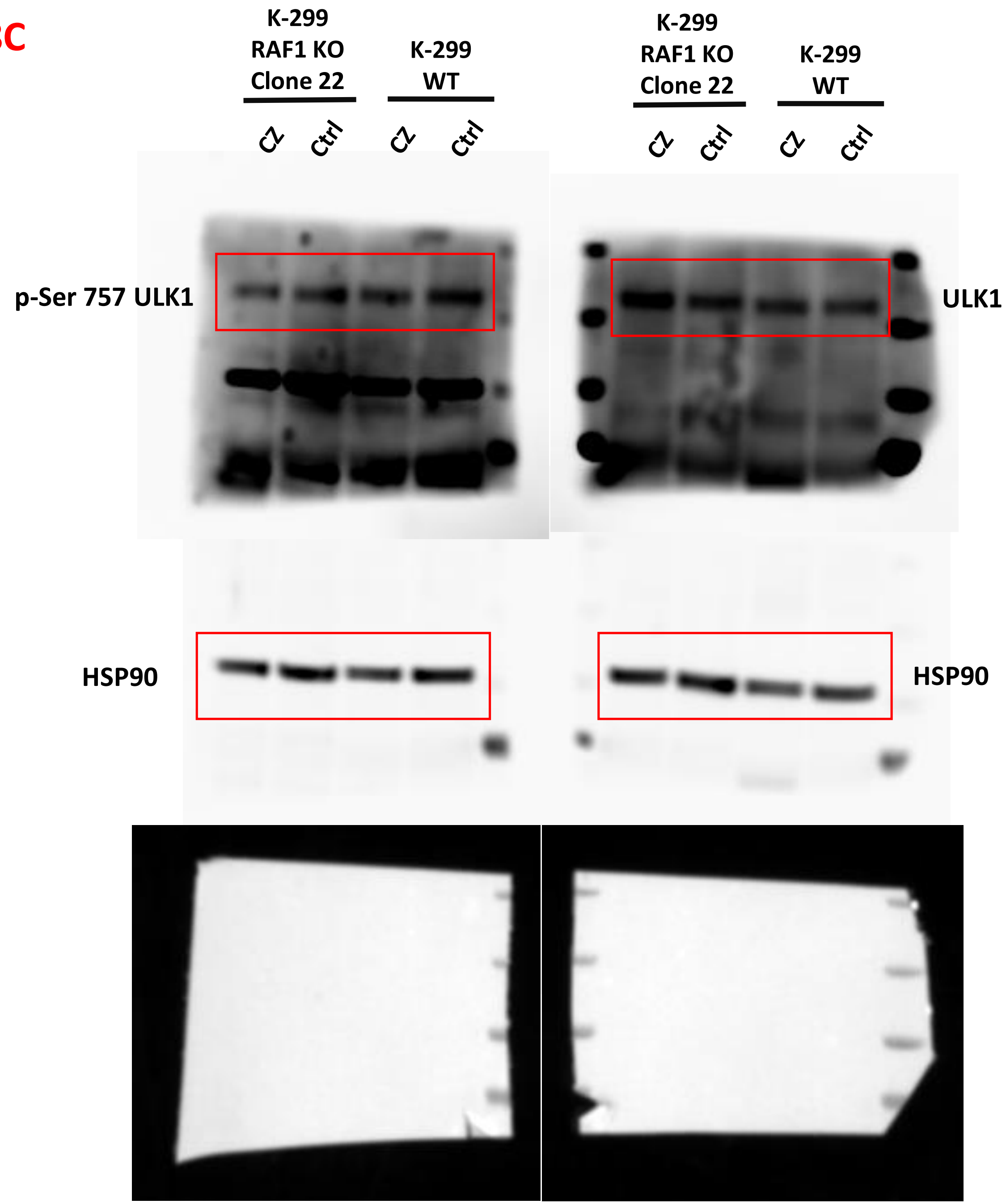

Full blots for Supplemental Figure 8D

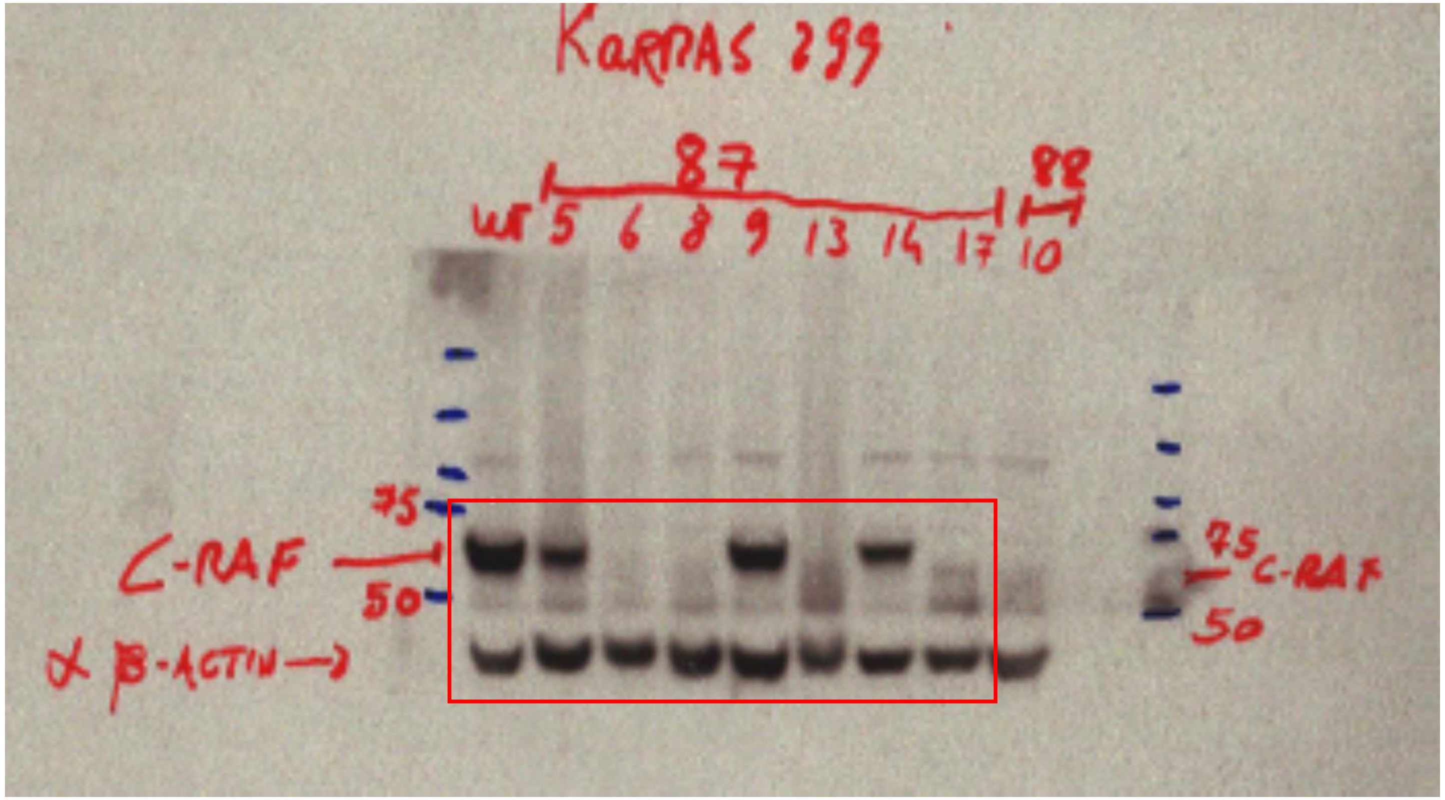

Supplement: Supplementary file 1 [file cancers-12-02951-s001.zip › cancers-967315-Supplementary Material File 2-original WB figures.pdf]
